# Supplementary material for: Vector Copy Distribution at a Single-Cell Level Enhances Analytical Characterization of Gene-Modified Cell Therapies
Source: Mol Ther Methods Clin Dev. 2020 Apr 25;17:944–56. doi: 10.1016/j.omtm.2020.04.016 (PMC7217927; doi:10.1016/j.omtm.2020.04.016)
Supplement: Document S1. Figures S1–S5 and Table S1 [file mmc1.pdf]

OMTM, Volume *17*

Supplemental Information

Vector Copy Distribution at a Single-Cell Level Enhances Analytical Characterization of Gene-Modified Cell Therapies

Ilaria Santeramo, Marta Bagnati, Emily Jane Harvey, Enas Hassan, Beata Surmacz-Cordle, Damian Marshall, and Vincenzo Di Cerbo

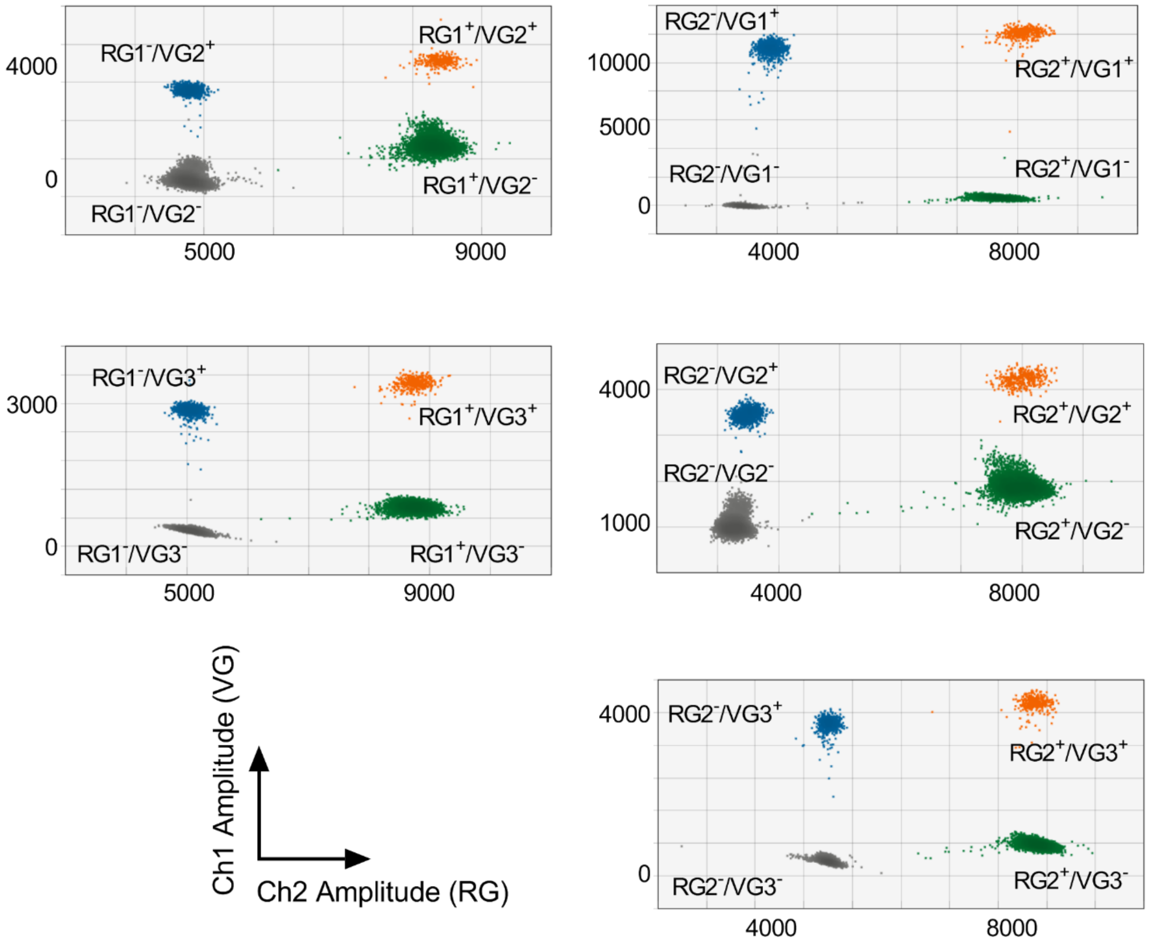

Figure S1 Representative 2D plot of duplex ddPCR reactions using different combinations of vector or human reference genes (see also Figure 1). Single positive droplets are shown in green for human targets (RG) and in blue for viral targets (VG), whereas double positive droplets are in orange and double negatives are in grey.

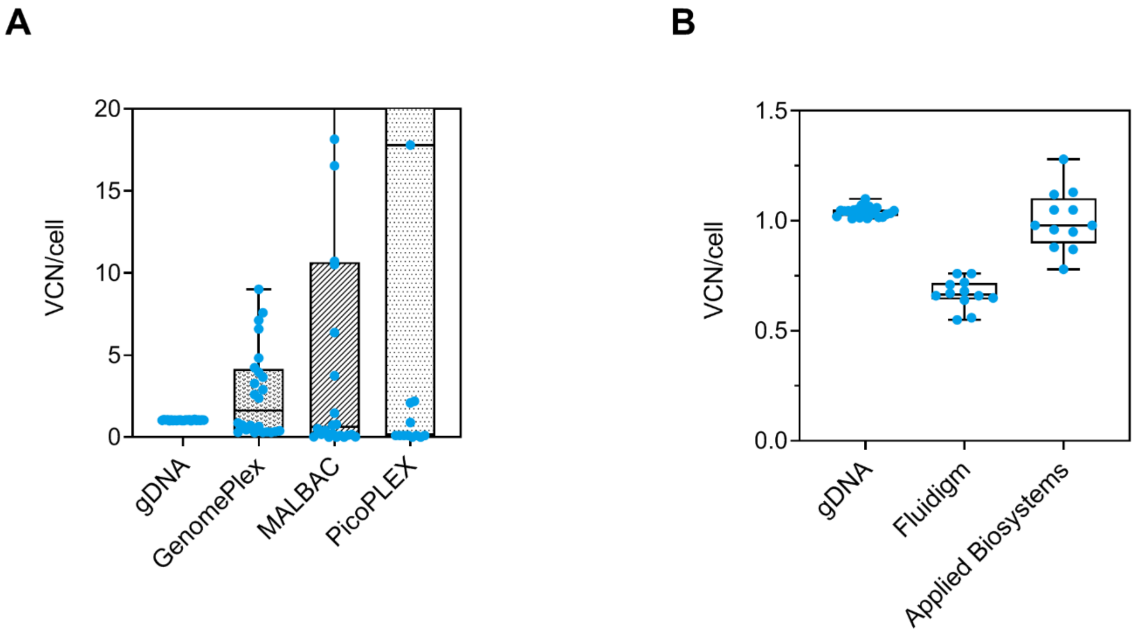

Figure S2 VCN analysis comparing WGA and targeted pre-amplification (see also Figure 2). (A) Summary of the vector copy number measured from each WGA reaction with the indicated commercial kits. Non-amplified genomic DNA is shown as control. Each boxplot (n=4, 24 data points) is represented with median and whiskers spanning from min to max value. For clarity, y-axis has been limited to 20 VCN/cell for an optimal visualisation of the data points closer to the gDNA positive control. (B) Summary of the vector copy number measured from each targeted pre-amplification reaction with the indicated commercial master mix. Non-amplified genomic DNA is shown as a control. Each boxplot (n=2, 12 data points) is represented with median and whiskers spanning from min to max value.

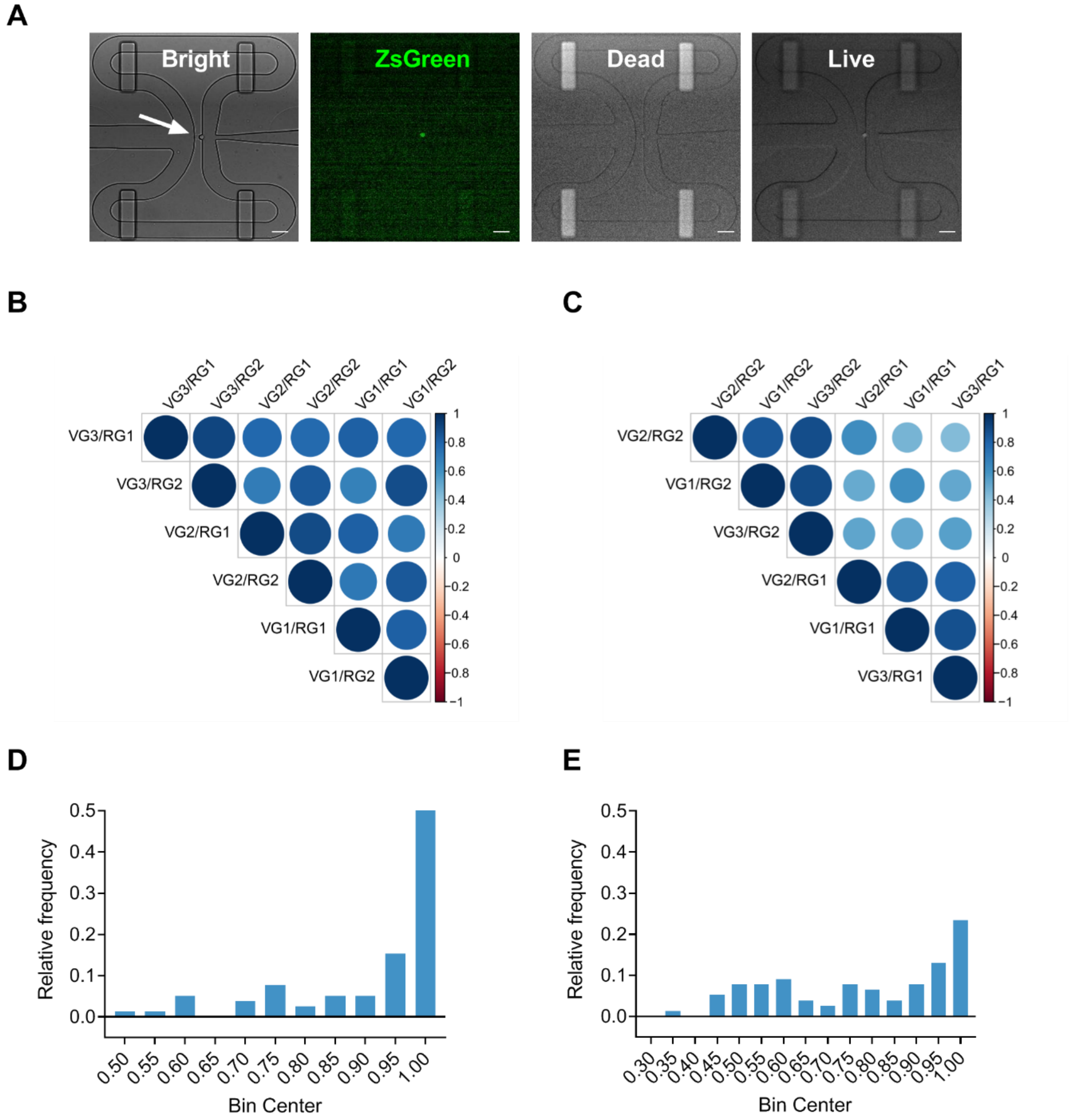

Figure S3 scVCN assay controls (see also Figure S3). (A) Representative images of one capture site in the Fluidigm OpenApp microfluidic chip. The arrow in the brightfield image indicates the capture site. Green signal derives from fluorescent ZsGreen protein. Live/dead detect the presence of calcein blue AM for live cells or ethidium homodimer-1 for dead cells (negative in this representative single cell). Scale bar = 60 µm. (B-C) Pairwise Spearman correlation between all duplex combinations on the “low VCN” (B) or the “high VCN” samples (C). Dark blue indicates positive correlation, whereas red indicates anti-correlation and the legend on the right shows the corresponding correlation coefficients. (D-E) Frequency distribution of highest posterior probabilities from the Bayesian analysis corresponding to the scVCN predictions with the highest likelihood. The probabilities for the low or high VCN samples are shown in (D) or (E), respectively. Bin width is 0.05.

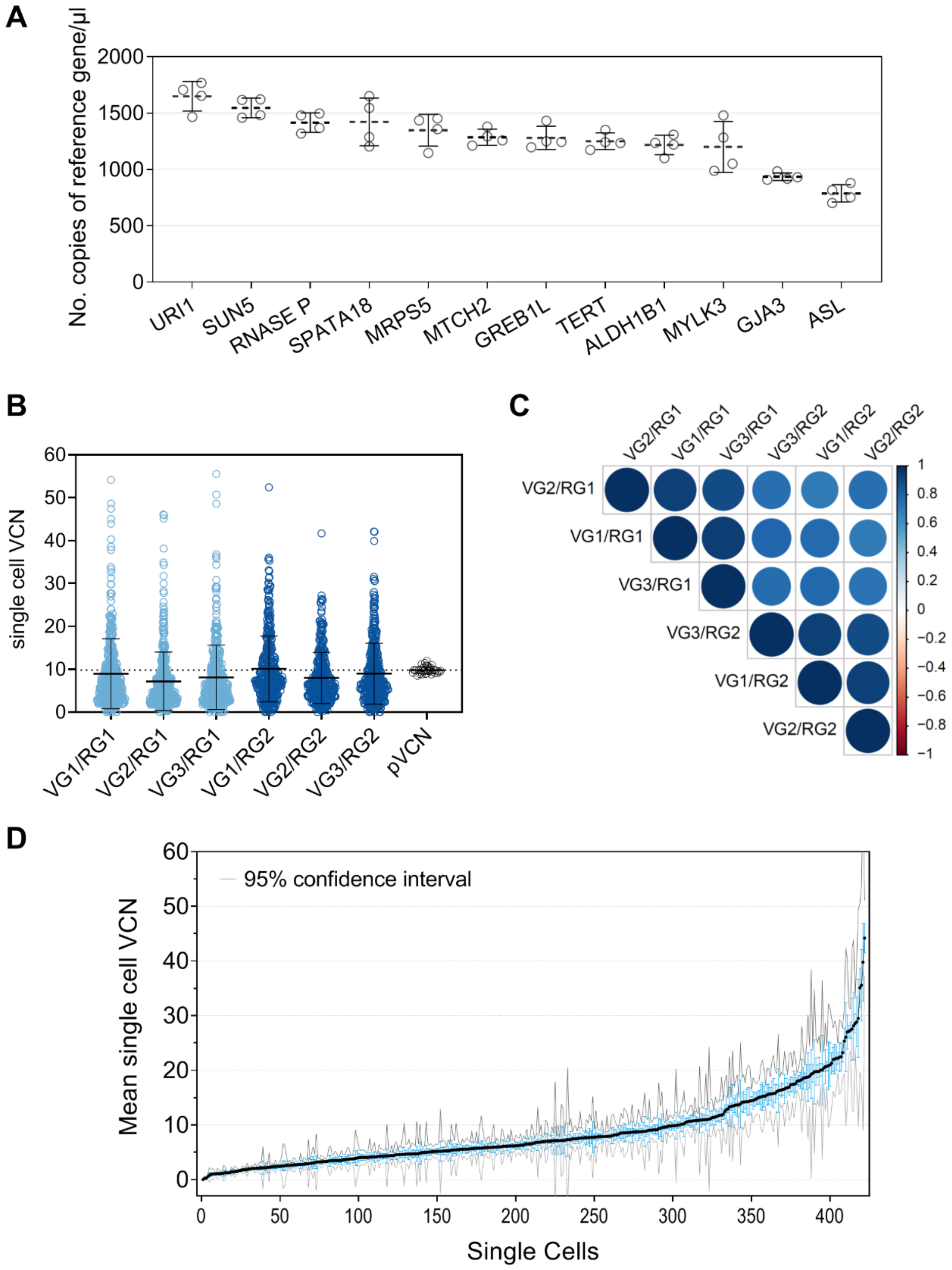

Figure S4 scVCN assay optimisation and scale out (see also Figure 4). (A) Absolute number of copies per µL of reaction of twelve reference genes tested on the genomic DNA equivalents of 80 cells pre-amplified with Fluidigm pre-amp mix (n=4). Each boxplot is represented with mean and standard deviation. (B) Single-cell VCN values from three triplex ddPCR assays (one VG and two RG in each reaction) generate six unique VG/RG measurements. scVCN values are in light blue for RG1 and in dark blue for RG2. pVCN from identical triplex ddPCR on bulk gDNA is shown in grey. Each boxplot is represented with mean and standard deviation. (C) Pairwise Spearman correlation between the six scVCN combinations across all six microfluidics chips. Dark blue indicates positive correlation, whereas red indicates anti-correlation and the legend on the right shows the corresponding correlation coefficients. (D) The mean of the six scVCN combinations is shown for each single cell and represented with standard error and 95% confidence interval. Single cells are ordered by increasing mean values.

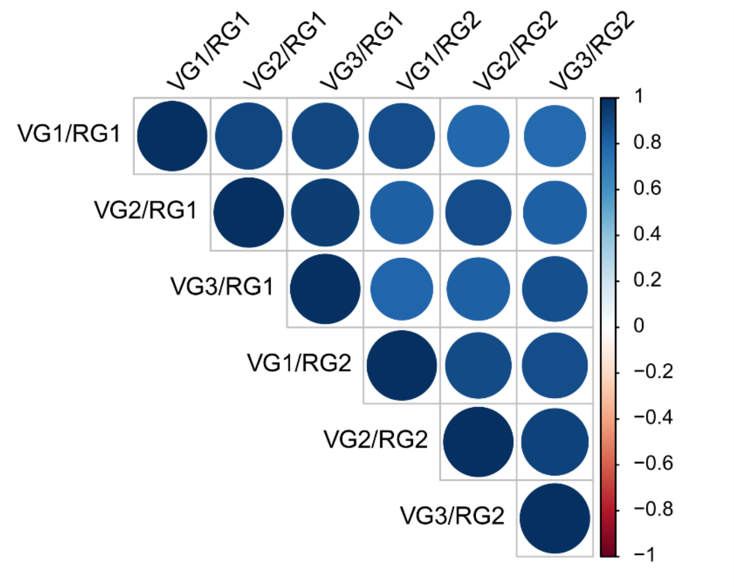

Figure S5. scVCN assay control for a non-sorted sample (see also Figure 5). Pairwise Spearman correlation between the six scVCN combinations. Dark blue indicates positive correlation, whereas red indicates anti-correlation and the legend on the right shows the corresponding correlation coefficients.

Table S1 VCN result summary of pre-amplification methods on gDNA (see also Figure 2).

	Mean	SD	CV
**GenomePlex**	2.66	2.71	102%
**MALBAC**	15.20	41.45	273%
**PicoPLEX**	918.70	2440.00	266%
**Fluidigm pre-amp mix**	0.67	0.07	10%
**Applied Biosystems pre-amp mix**	1.00	0.13	13%
**gDNA (non-amplified)**	1.04	0.02	2%
