# Supplementary material for: Vector Copy Distribution at a Single-Cell Level Enhances Analytical Characterization of Gene-Modified Cell Therapies
Source: Mol Ther Methods Clin Dev. 2020 Apr 25;17:944–56. doi: 10.1016/j.omtm.2020.04.016 (PMC7217927; doi:10.1016/j.omtm.2020.04.016)
Supplement: Document S2. Article plus Supplemental Information [file mmc2.pdf]

Molecular Therapy
Methods & Clinical Development
Original Article

Vector Copy Distribution at a Single-Cell Level Enhances Analytical Characterization of Gene-Modified Cell Therapies

Ilaria Santeramo,[1] Marta Bagnati,[1] Emily Jane Harvey,[1] Enas Hassan,[1] Beata Surmacz-Cordle,[1] Damian Marshall,[1] and Vincenzo Di Cerbo[1]

[1]Cell and Gene Therapy Catapult, 12th Floor Tower Wing, Guy's Hospital, Great Maze Pond, London SE1 9RT, United Kingdom

The ability to deliver transgenes into the human genome using viral vectors is a major enabler of the gene-modified cell therapy field. However, the control of viral transduction is difficult and can lead to product heterogeneity, impacting efficacy and safety, as well as increasing the risk of batch failure during manufacturing. To address this, we generated a novel analytical method to measure vector copy distribution at the single-cell level in a gene-modified, lentiviral-based immunotherapy model. As the limited amount of genomic DNA in a single cell hinders reliable quantification, we implemented a preamplification strategy on selected lentiviral and human gene targets in isolated live single cells, followed by quantification of amplified material by droplet digital PCR. Using a bespoke probability framework based on Bayesian statistics, we show that we can estimate vector copy number (VCN) integers with maximum likelihood scores. Notably, single-cell data are consistent with population analysis and also provide an overall measurement of transduction efficiency by discriminating transduced (VCN $\geq$ 1) from nontransduced (VCN = 0) cells. The ability to characterize cell-to-cell variability provides a powerful high-resolution approach for product characterization, which could ultimately allow improved control over product quality and safety.

INTRODUCTION

Gene-modified cell therapies have the potential to circumvent pathological conditions caused by genetic aberrations by introducing exogenous therapeutic transgenes into host cells. Unlike standard treatments using small-molecule drugs or biopharmaceuticals, which are designed to prevent or manage disease progression, cell and gene therapies often have long-lasting curative outcomes. This creates a new way to control disease and has fueled a rapidly growing and evolving field. In the past 5 years, there have been 11 new therapies approved by the U.S. Food and Drug Administration (FDA) and/or European Medicines Agency (EMA),[1–3] and there are over 1,000 clinical trials currently being performed globally.[4] Key to the success of this field has been the use of viral vectors that are the preferred delivery system for both gene therapies and gene-modified cell therapies to endow cells with functional copies of otherwise mutated genes or with synthetic genetic elements that exert novel biological functions. The ease with which their genome can be engineered and the relatively large cargo (up to 5 kb) they can accommodate have allowed their extensive use in more than 70% of current clinical trials.[4] Vectors belonging to the retroviridae family, such as retroviruses and lentiviruses, can stably integrate into the host genome, providing potential long-term therapeutic benefits. However, these advantages are tempered by the intrinsic risk of insertional mutagenesis, which may occur when viral integration impairs the functionality of proto-oncogenes.[5–9] To address concerns about these risks, regulatory authorities require cell therapy products utilizing viral transduction to undergo monitoring and reporting of various product specifications, including number of vector integrations per cell and transduction efficiency.[10,11]

The standard approach for measuring vector copy number (VCN) is through population analysis. In this approach, genomic DNA (gDNA) is extracted from bulk cells, and the total number of viral genomes, as determined by quantitative PCR (qPCR), represents the average of the whole population. However, as this approach is based on bulk DNA, it does not give a reliable representation of the true number of vector integrations in each cell nor the underlying cell-to-cell variability in the distribution of vector copies (Figure 1A). This may have implications for product safety, as it may underestimate the presence of cell clones with a high number of integrations that could persist and replicate following cellular transplantation.[12–14] It may also lack the resolution to pinpoint changes in the final product specifications due to intrinsic variability in the manufacturing process caused, for instance, by the patient-specific donor cell material or lot-to-lot variability of vector batches.[15,16] Overcoming the disadvantages of population VCN (pVCN) could be achieved by measuring viral vector integrations in individually isolated single cells.[13,17–19] Single-cell methods have been largely employed to discern the composition of cell populations[20,21] by various transcriptomic and/or proteomic approaches,[22–25] whereas novel methods that

Received 2 April 2020; accepted 22 April 2020;
https://doi.org/10.1016/j.omtm.2020.04.016.

Correspondence: Vincenzo Di Cerbo, Cell and Gene Therapy Catapult, 12th Floor Tower Wing, Guy's Hospital, Great Maze Pond, London SE1 9RT, UK.
E-mail: vincenzo.dicerbo@ct.catapult.org.uk

 © 2020 The Authors.
This is an open access article under the CC BY license (http://creativecommons.org/licenses/by/4.0/).

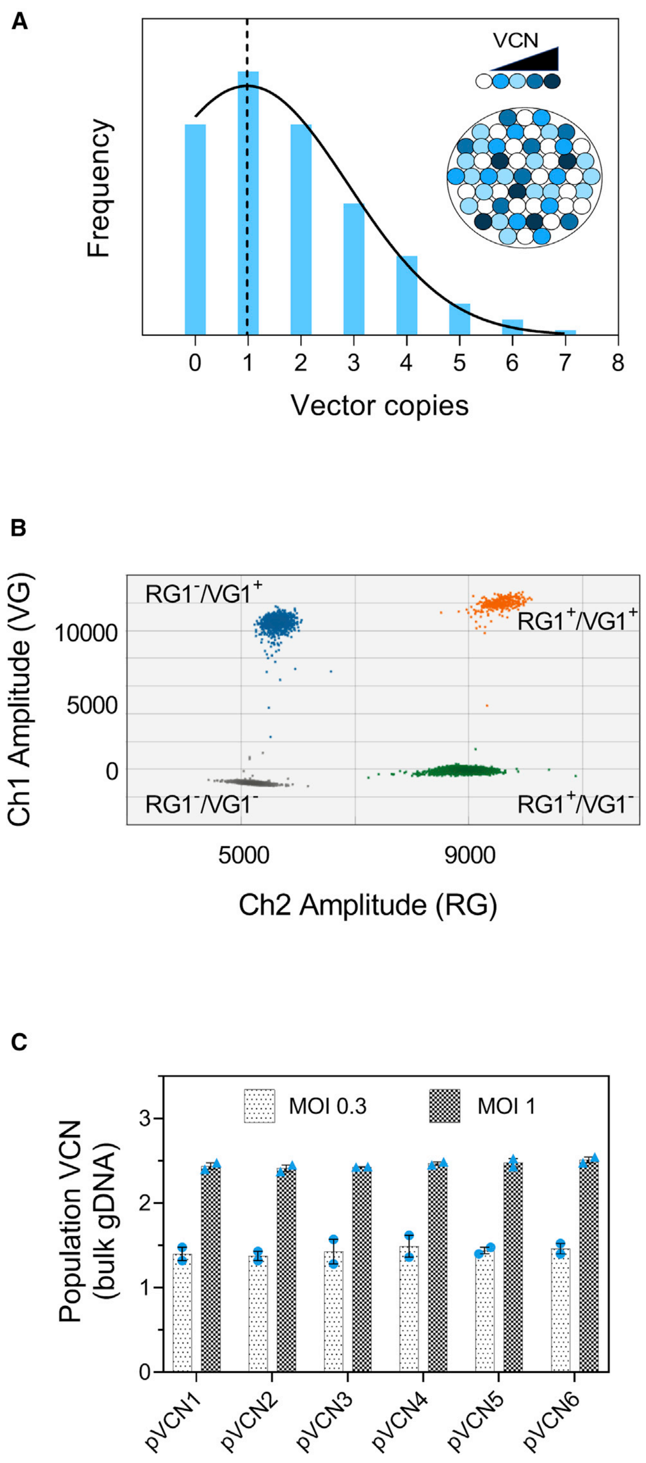

Figure 1. Population Vector Copy Number Analysis by ddPCR

(A) Population average (dashed line) can underlie a broad VCN distribution at a single-cell level, and this can be indicative of the population heterogeneity (inset). (B) Representative 2D plot of a duplex ddPCR reaction with VG1 and RG1 targets. Single positive droplets are shown in green for human targets and in blue for viral targets, whereas double-positive droplets are in orange and double negatives are in gray. (C) ddPCR analysis of the population VCN in cells transduced at MOI 0.3 or MOI 1. Two independent biological replicates (blue dots) for each condition were subject to duplex ddPCR reactions using unique combinations of vector and human reference assays for a total of six different measurements (pVCN1–6). Error bars indicate ranges. VG, vector gene; RG, human reference gene. See also Figure S1.

encompass analysis of additional genetic and epigenetic features are constantly developed.[26–30] However, to date, these methods have largely been used to measure nucleic acid or protein targets that are present at relatively high levels. Consequently, the sensitivity of single-cell analysis for detection of single-copy targets, such as vector integrations, is poorly explored.

To overcome this limitation, droplet digital PCR (ddPCR) technology offers a valuable solution, given its ability to provide absolute quantification of low abundant targets and rare events. In contrast to the conventional qPCR methodology, which is influenced by the efficiency of the primers to anneal and linearly amplify their target sequences and the requirement for standards at known concentrations to allow relative quantification, ddPCR does not required reference standard curves or linear amplification kinetics.[31,32] Instead, the sample for analysis is partitioned into thousands of individual reactions using a water-oil emulsion-droplet technology. An end-point PCR reaction is performed to provide a digital fingerprint of positive and negative droplets, which in turn, provides the absolute quantification of the targets based on Poisson statistics. This approach is increasingly used within the field to measure, for example, viral titer based on total genome copies.[33–37] However, to allow for a more reliable quantification, tens to hundreds of copies of a target are preferable, which may limit the direct application of this method to single-cell analysis. Therefore, a ddPCR approach would benefit from the development of robust techniques for unbiased preamplification of single-cell genomic material to allow quantification of individual vector integrations into the host genome on a cell-by-cell basis.

In this study, we use a human primary T cell immunotherapy model transduced with a lentivirus encoding a green fluorescent protein (ZsGreen) to design a high-content, single-cell method for measuring vector copy number. Using ddPCR, we apply a multiplexing strategy to quantify simultaneously lentiviral vector and human reference sequences in the same reaction. We then evaluate the performance of a range of reagents for preamplification of DNA prior to ddPCR analysis. By selecting the best nonbiased preamplification approach, we then demonstrate how vector and human genomic sequences can be measured by ddPCR in individually isolated cells and the single-cell VCN (scVCN) estimated down to single copy number level by a Bayesian statistics framework. This novel, highly sensitive approach for single-cell VCN provides, for the first time, a rapid and robust tool to investigate cell-to-cell variations during viral transduction. We anticipate that the detailed characterization of gene-modified cell therapies will allow tighter control over product safety, as well as increased understanding of product variability.

RESULTS

Population VCN Measurements Using Droplet Digital PCR

Human peripheral blood mononuclear cells (PBMCs) were derived from healthy donors, and cluster of differentiation (CD)3$^+$ T cells were purified using magnetic separation. Preactivated T cells were transduced using a commercially available HIV-1 lentivirus encoding a green fluorescent ZsGreen protein at a multiplicity of infection (MOI) of either 0.3 or 1.0. Cells were cultured for 7 days, after which, transduction efficiency was measured by flow cytometry, yielding an average transduction level between 30% and 50% (data not shown). To measure the VCN of the transduced cells at the pVCN level, we designed three hydrolysis-probe assays targeting *RRE* (VG1), *WPRE* (VG2), and the *ZsGreen* transgene (VG3) vector sequences. These were used in combination with two commercially available human copy number reference gene (RG) assays for ribonuclease P RNA component H1 (*RPPH1*; RG1) and telomerase reverse transcriptase (*TERT*; RG2) to generate six unique combinations of duplex ddPCR reactions (Figures 1B and S1). The analysis showed that the mean and standard deviation for each pVCN was 1.43 ± 0.10 (MOI 0.3) and 2.45 ± 0.05 (MOI 1.0), indicating a 1.7-fold increase in vector copies per cell when increasing the vector load (Figure 1C). The coefficient of variation (CV) for all of the six combinations of primers was 7% (MOI 0.3) and 2% (MOI 1.0) with CVs within each primer pair ranging between 4% and 4.6%. These values are comparable to accepted ddPCR intra-assay variations for identical replicates,[38,39] suggesting that each duplexed assay is equally effective at measuring vector copies at the population level.

Optimal Preamplification of Genomic DNA Is Achieved with Targeted Amplification

In order to apply the established ddPCR bulk assays to single cells, genomic targets required an initial round of preamplification to generate sufficient input material for accurate quantification of multiple targets over background noise. To ensure that preamplification can be achieved without introducing a PCR bias, we tested a range of commercial kits for both whole genome amplification (WGA; three kits: PicoPlex, GenomePlex, and Multiple Annealing and Looping Based Amplification Cycles [MALBAC]) or targeted amplification (two kits: Fluidigm and Applied Biosystems) and assessed whether there was a proportional amplification across several targets. For these initial optimization experiments, gDNA was extracted from bulk transduced cells and used to test kit performance following the manufacturers' protocols.

For each WGA kit, four gDNA samples were subjected to parallel, independent reactions, and each sample was assayed with the six duplexed ddPCR primer combinations previously established. Absolute quantification of the number of amplified target copies showed a large amplification bias for both the vector genes (VGs) and human RGs for all target sequences (Figure 2A). In particular, RG2 failed to be successfully amplified in all three kits (10- to 1,000-fold difference to RG1), whereas VGs were amplified nonuniformly with the mean CV of 42%, 140%, and 129% for GenomePlex, MALBAC, and PicoPlex, respectively. Variability among the technical replicates was also observed within each target for both PicoPlex and MALBAC kits, with CVs ranging between 21%–84% and 30%–78%, respectively, whereas GenomePlex performed more consistently and showed CVs between 5% and 29%. Notably, nonamplified gDNA used as a control showed no differences within the groups of genes (Figure 2B) and inter-replicate variation of around 2%–3%, demonstrating that the bias observed with the WGA kits was independent of the ddPCR readout. Consequently, when average VCN was calculated from these WGA data, mean and CV values were too high to be considered accurate (Figure S2A; Table S1).

For targeted preamplification, we tested two commercially available master mixes by Fluidigm or Applied Biosystems adapting the manufacturers' instructions to gDNA amplification. Two gDNA replicates were analyzed by ddPCR using the six duplex hydrolysis-probe assay combinations for each sample. Following targeted preamplification, the resulting absolute number of copies was very consistent within each group of genes with mean variations in the VG group of 9% for the Fluidigm mix or 20% for the Applied Biosystems one and mean differences in the RG group of 11% and 14% for the Fluidigm and the Applied Biosystems mixes, respectively (Figure 2C). Moreover, the calculated VCN of 1.00 and 0.67 for Applied Biosystems and Fluidigm preamplification mixes (CV 13% and 10%), respectively (Figure S2B), was consistent with the nonamplified gDNA VCN of 1.00 (Table S1). These results provided sufficient evidence that the targeted approach leads to linear preamplification of the target genes and therefore, was used as the preamplification method for subsequent experiments on single cells.

Single-Cell Analysis Provides a Vector Copy Number Distribution Consistent with Population Measurements

To test the application of preamplification and ddPCR for single-cell analysis, we initially fluorescence-activated cell sorted (FACS) two samples of independently transduced cells with different bulk pVCN levels and selected highly pure ZsGreen$^+$ transduced populations. Samples from the sorted cell populations underwent gDNA extraction, were analyzed using the six duplex ddPCR assays, and were shown to have a pVCN of 1.46 ± 0.32 ("low") and 2.93 ± 0.12 ("high"), respectively. Concomitantly, each sorted cell sample was loaded onto a Fluidigm C1 Single-Cell AutoPrep System to capture individual cells (Figure 3A), which were then stained with calcein blue acetoxymethyl (AM; live cell dye) and ethidium homodimer-1 (nonviable cell dye). The microfluidic chip was imaged to identify capture sites containing one live cell (Figure S3A) and showed an overall capture rate of live single cells of 75%. The captured ZsGreen$^+$ cells underwent gDNA extraction and targeted preamplification within the microfluidic chip, and the amplified material was analyzed by duplex ddPCR reactions to generate six single-cell VCN datasets from each unique duplex combination (Figures 3B and 3C). This showed that although the average VCNs for each duplex combination were comparable to each other and to the bulk gDNA, there was a high level of heterogeneity in VCNs at the single-cell level, information that was unavailable in the population gDNA analysis. Comparison between each dataset showed small

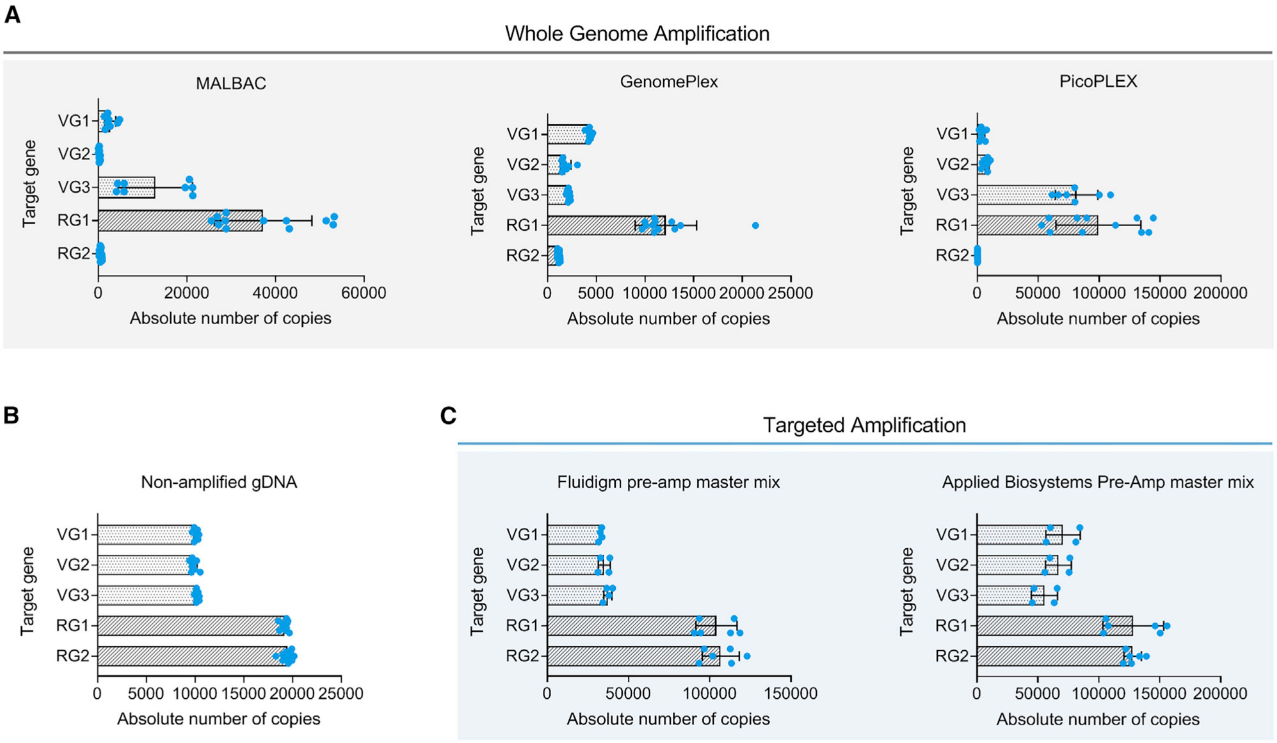

Figure 2. Targeted Preamplification, but Not WGA, Follows Linear Amplification Kinetics and Maintains the Relative Abundance of the Target Genes
(A) Absolute number of target-gene copies recovered by ddPCR after WGA reactions with three commercial kits across four parallel and independent WGA reactions. (B) Nonamplified gDNA is used as a ddPCR control and shows a uniform absolute number of target-gene copies across four gDNA samples. (C) Absolute number of target-gene copies recovered by ddPCR after targeted preamplification reactions with two commercial master mix reagents across two parallel and independent reactions. Three vector genes (VG1–3) are indicated by clear bars; two human reference genes (RG1 and RG2) are indicated by dark bars. Each blue dot represents one measurement from an independent duplex ddPCR reaction. Error bars show the standard deviation. VG1, RRE; VG2, WPRE; VG3, ZsGreen; RG1, RPPH1; RG2, TERT. See also Figure S2.

variations in VCN measurement from the same single cell, and this was globally monitored by pairwise correlations, which exhibited an overall positive correlation between each combination (Figures S3B and S3C) with a mean Spearman correlation coefficient of 0.779 for the low pVCN sample and 0.641 for the high pVCN sample. This suggested that the presence of minor variations was likely due to the use of diverse and unique combinations of target genes instead of identical technical repetitions of the same target combination. Next, for each individual cell, we calculated the mean scVCN from the six ddPCR measurements and used the standard error of the mean to visualize the technical variability (Figures 3D and 3E). As expected, the mean value provides a measurement for scVCN that is not an integer, which creates an uncertainty in the exact scVCN estimate. To account for this uncertainty, we generated a computational framework based on Bayesian statistics to model the probability of each possible copy number. For each individual single cell, the copy with the maximum probability was derived to estimate the scVCN integer per cell with the highest likelihood score (Figures S3D and S3E). With the use of this statistical framework, we derived scVCN predictions and grouped these to express the proportion of each copy number per cell in the population (Figures 3F and 3G). This approach allows the evaluation of the heterogeneity of the VCN distribution at the single-cell level in the transduced cell populations. The sample with an average pVCN of 1.46 vector copies per cell contained approximately 64% of cells with one copy, 23% with two copies, 5% with three copies, and 3% with four copies. No cells were detected that had five or more copies (Figure 3F). Notably, for the sample with a higher average pVCN of 2.93, the distribution of vector copies shifted toward higher values, with only 27% of single cells having one copy, 40% of cells with two copies, and 18% with three copies, whereas 9% of cells had five or more vector copies (Figure 3G).

Performance of the scVCN Assay and Further Optimization

Single-cell vector copies may be collated together to provide a mean of predictions that can be compared to the pVCN value to evaluate the consistency of the method against a reference value and highlight potential sampling effects under the assumption that the measured pVCN corresponds to the true population VCN. We observed that the mean of scVCN predictions and the pVCN were highly comparable, with differences (ΔVCN) of 2.0% and 19.8% for the low and high pVCN samples, respectively (Figures 3F and 3G, bottom).

In order to gain further understanding of the performance of the assay, we applied the scVCN workflow to seven additional sorted transduced samples and observed that populations characterized by

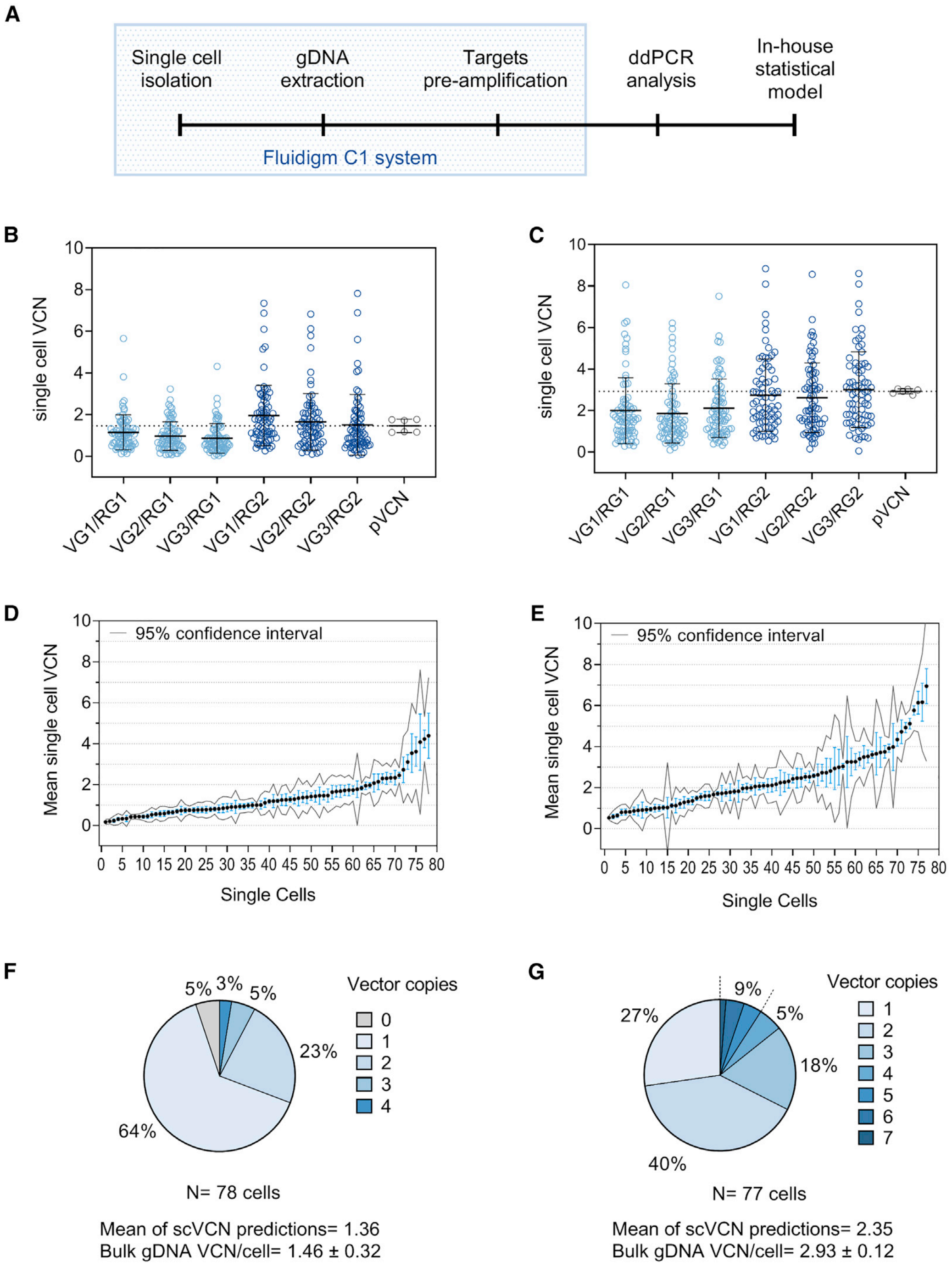

Figure 3. Single-Cell Vector Copy Number Assay on Two Cell Samples

(A) Diagram of the scVCN workflow. The steps in the light blue box are performed within the closed Fluidigm C1 system. ddPCR analysis is performed on each single-cell preamplified material on the QX200 Bio-Rad system. (B and C) Single-cell VCN values from each duplex assay combination are shown for the low (B) or the high (C) pVCN samples, whereas the values from bulk gDNA analysis (gray) include all six combinations and are shown as reference. Combinations originating from RG1 are in light blue, whereas the combinations with RG2 are in dark blue. Each boxplot is represented with mean and standard deviation. (D and E) The mean of the six scVCN combinations is shown for each single cell in the low (D) and high (E) pVCN samples and represented with standard error and 95% confidence interval. Single cells are ordered by increasing mean values. (F and G) Proportion of single cells with predicted vector copy units determined by Bayesian analysis in the low (F) and high (G) pVCN samples. The percentage of single cells with five or more vector copies is grouped. pVCN from bulk population analysis is indicated at the bottom with the standard deviation, alongside the mean of the single-cell VCN predictions. See also Figure S3.

(legend continued on next page)

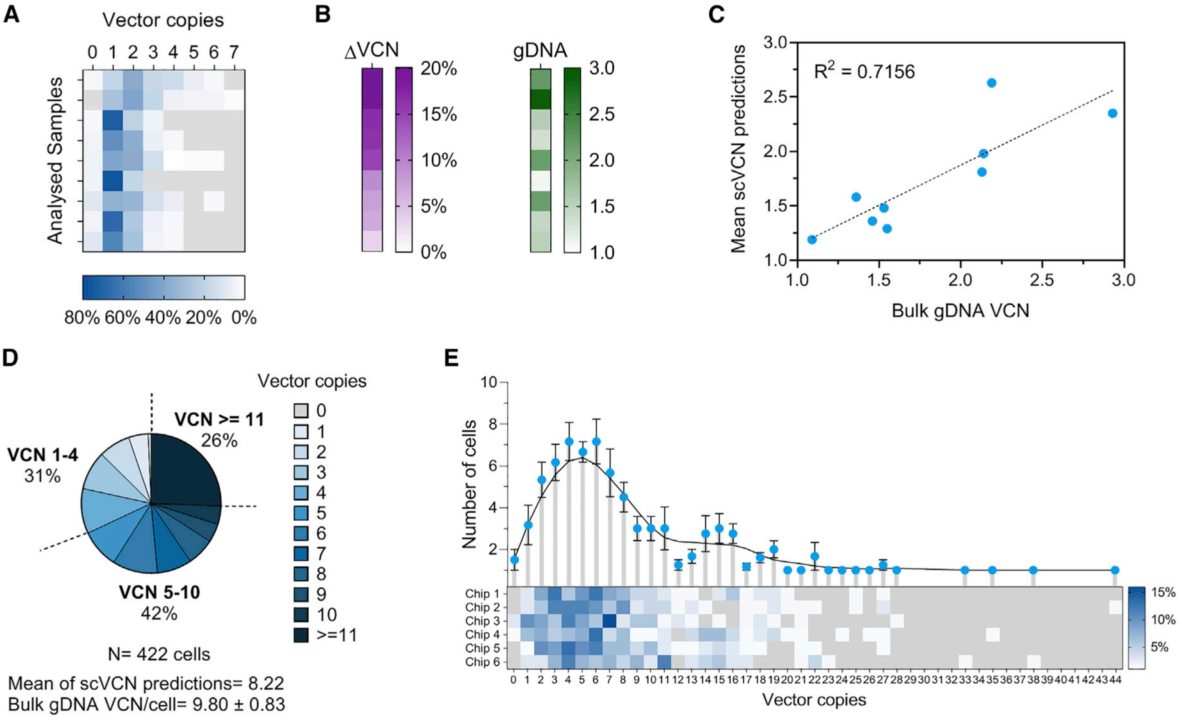

Figure 4. Performance of the scVCN Assay

(A) scVCN distribution in nine FACS-transduced cell samples (blue heatmap). The blue gradient represents the proportion (%) of single cells with the indicated number of vector copies in each sample. Vector copies not detected in the samples are in light gray. (B) Heatmaps of the ΔVCN (purple), which is the difference (%) between the mean of the scVCN predictions and the bulk VCN (green). Samples in both heatmaps are ordered by decreasing ΔVCN values. (C) Linear regression between population VCN and mean of the scVCN predictions showing a positive correlation (R^2 = 0.7156). (D) Proportion of single cells (%) with predicted vector copy integers. For representation purposes, single cells with 11 or more copies are grouped, and percentages are shown as cumulative values of three VCN ranges. Corresponding pVCN from bulk gDNA ddPCR analysis is indicated at the bottom with the standard deviation, alongside the mean of the single-cell VCN predictions. (E) A sample with pVCN = 9.80 was analyzed across six Fluidigm chips. The variability in the scVCN distribution for each chip is showed in the heatmap (bottom), whereas the average number of cells within each VCN level is represented (mean ± SEM) with a locally weighted scatterplot smoothing (LOWESS) fit (top). p value > 0.05. See also Figure S4.

a higher bulk VCN generally showed a wider range of vector integrations (Figures 4A and 4B). Moreover, whereas the difference from the population VCN value seemed greater in some individual samples with high pVCN, we did not observe any clear trend when comparing side-by-side ΔVCN and bulk gDNA (Figure 4B). Notably, this difference was consistently less than or equal to ~20% (Figure 4B); thus, we deemed these ΔVCN values acceptable for such type of single-cell prediction and expected the overall accuracy of the method to be equal to or greater than 80% under these experimental conditions. To corroborate these observations, linear regression analysis demonstrated that the mean scVCN correlated positively with the pVCN (R^2 = 0.7156) with a nonsignificant deviation from linearity (Figure 4C).

As these initial studies were performed on a small population size using one single Fluidigm chip, we sought to increase the throughput of the analysis and test the feasibility of the method when analyzing multiple chips per sample. To facilitate this analysis, we optimized the ddPCR readout by introducing minor modifications to the multiplexing strategy and the primer design, whereas the complete workflow for single-cell isolation, processing, and data analysis was unchanged. We initially screened a larger panel of commercially available human reference genes during preamplification experiments on gDNA and compared these with the RG1 (*RPPH1*) and RG2 (*TERT*) human targets (Figure S4A). As *RPPH1* and *SPATA18* showed similar preamplification levels, we selected these two for ddPCR analysis. Additionally, to extend the applicability of the method to other types of lentiviral-based samples, one of the targets specific for the *ZsGreen* gene cargo (VG3) was replaced by primers targeting a conserved region on the lentiviral backbone[40] within the *Psi* sequences, which also maintained similar preamplification levels with the other VG targets (data not shown). These primers were tested in duplex and triplex reactions (i.e., using one vector gene and two reference genes in combination) and showed no difference in ddPCR quantification (data not shown).

Using these optimized experimental conditions, we set out to verify whether the single-cell VCN distribution was a bona fide representation of the whole population by analyzing independent samplings of the same population and ultimately to increase the total sample size.

Given that the previously analyzed samples had a bulk VCN range of 1–3 and thus provided relatively narrow scVCN distributions, we sought to increase purposely the population heterogeneity by generating a transduced sample with bulk VCN of 9.80 ± 0.83, expecting to obtain a substantially wider VCN range that could reveal putative subsampling variations. For this experiment, we used six microfluidics chips to isolate a total of 422 live single-transduced cells (73% average single-cell recovery) for which we observed a broad range of ddPCR vector copy measurements (Figure S4B). Notably, the new primer combinations encompassing VG3 and/or RG2 showed highly similar results for single-cell VCN values in comparison to the unmodified combinations (Figure S4B) and an improved pairwise correlation with a mean Spearman coefficient of 0.800 (Figure S4C). Next, we determined the mean VCN for each individual cell and estimated the technical variability among the six primers/probe combinations (Figure S4D). The scVCN predictions that were generated with the Bayesian model confirmed the high heterogeneity of the sample and showed vector copies spanning from 1 to 44. Thus, we grouped the single cells by VCN ranges to reflect supposedly decreasing safety levels and reported 31% of cells in range 1–4, 42% in range 5–10, and 26% of single cells with 11 or more vector copies (Figure 4D). Notably, with the comparison of the mean of the single-cell VCN predictions to the corresponding bulk pVCN, we observed a 16% difference, which was similar to the variation observed for all of the other samples with bulk pVCN between 1 and 3, corroborating the reliability of the assay for samples with substantially higher bulk pVCN. Next, we segregated the single cells by the microfluidic chip on which they were isolated, and we analyzed each dataset independently but with the same quality thresholds to evaluate the effect of subsampling. Interestingly, we observed that despite the small population of single cells isolated on each chip (~70 cells on average) and the high heterogeneity of the sample, the distribution of the scVCN was markedly similar across all chips ($p > 0.05$, Kruskal-Wallis test), with the highest frequency of single cells bearing 3 to 7 vector copies (Figure 4E). Further pairwise comparison between chip combinations indicated a nonsignificant difference between each chip, although only chip 6 showed a minimal significant difference against chip 3, with a p value of 0.0437 (Kruskal-Wallis with Dunn's test multiple comparisons correction), yet nonsignificant differences with all other four chips (Figure 4E). Therefore, these results indicated the absence of significant subsampling effects in a sample with a bulk VCN of ~10 and suggested that small sample sizes could reliably represent the distribution of the vector copies in the population of transduced cells.

Single-Cell VCN Analysis for a Lentiviral-Based Cell Therapy Product

As we replaced the *ZsGreen* primers/probe with new ones targeting the lentiviral backbone, we set out to use the assay on a broader range of lentiviral-based cell therapy products. For this, we tested the performance of the assay on a nonsorted transduced cell sample, which would more closely mimic gene-modified cell therapy medicines. Having established that scVCN distributions are consistent across multiple population samplings, we performed the analysis on two consecutive microfluidic chips to balance the expected reduction in the number of captured transduced cells due to the presence of nontransduced cells. The single-cell capture rate was 81% for chip 1 (N = 78) and 86% for chip 2 (N = 83), which provided a total of 161 single live cells. These underwent targeted preamplification within the Fluidigm C1 system and were then analyzed using the newly optimized ddPCR workflow, as previously described. Also, we confirmed with this analysis that the new primer/probe combinations encompassing VG3 and/or RG2 were similar to the unmodified combinations (Figure 5A), and this was supported by a good pairwise correlation with a mean Spearman coefficient of 0.857 (Figure S5). Next, for each individual cell, we calculated the mean scVCN from the six ddPCR measurements and used the standard error of the mean to visualize the technical variability (Figure 5B). As anticipated, the ddPCR analysis was able to detect nontransduced cells that had a normal signal for the human reference genes but no signal for the vector targets (i.e., scVCN = 0; Figure 5B), thus indicating no lentiviral integration. Statistical analysis using the Bayesian framework showed that 18% of cells had no vector integrations, followed by 29%, 22%, 17%, and 6% for one, two, three, and four vector integrations, respectively (Figure 5C). Around 8% of the cells had more than five vector integrations, with the highest scVCN being nine. The mean of the scVCN predictions was 1.9 copies per cell and was comparable with the pVCN of 2.3 ± 0.24 measured using genomic DNA with a ΔVCN of 17%, which is consistent with the previously observed values (Figure 4B). As the data were generated from the analysis of two microfluidic chips, we compared the number of observed events on each chip to verify whether the two scVCN distributions were consistent when considered as technical replicates. To do this, we analyzed each dataset independently, applying the same quality criteria, and produced two scVCN distributions (Figure 5D). Only minor differences were observed in the number of single cells with two to four copies, and statistical analysis confirmed that there was no significant difference between the two chips ($p = 0.9842$, two-tailed Mann-Whitney test).

Finally, as the sample contained a mixture of transduced and nontransduced cells, we compared the transduction efficiency value obtained by single-cell ddPCR with live imaging and flow cytometry. A direct comparison of these different methods produced similar values, with an overall transduction efficiency of ~80% (Figure 5E), suggesting that this method could provide a reliable PCR-based estimation of transduction efficiency.

DISCUSSION

Cell therapy products need to conform to a regulatory requirement prior to being released for infusion to patients. Therefore, tightly controlled processes are required to ensure these products meet rigorous standards for quality and safety. This is achieved during development and manufacturing through analytical characterization, which is pivotal for monitoring product specifications and critical quality attributes. However, as cell-based therapies are extremely complex to develop and highly susceptible to multiple sources of variability,[16,41,42] there is a growing need for advanced analytical tools

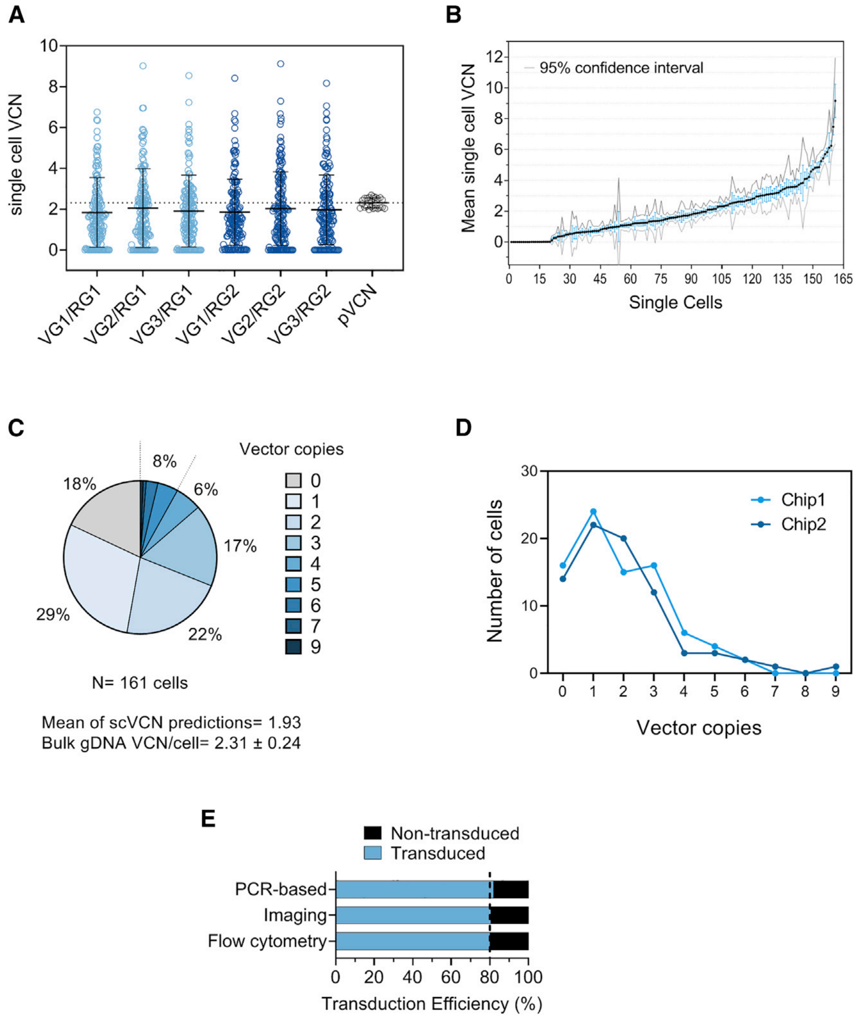

Figure 5. The scVCN Workflow Applied to Nonsorted Samples

(A) Single-cell VCN values from three triplex ddPCR assays (one VG and two RG in each reaction) generate six unique VG/RG measurements. scVCN values are in light blue for RG1 and in dark blue for RG2. pVCN from identical triplex ddPCR on bulk gDNA is shown in gray. Each boxplot is represented with mean and standard deviation. (B) The mean of the six scVCN combinations is shown for each single cell and represented with standard error and 95% confidence interval. Values with zero mean correspond to nontransduced single cells in which only reference genes, but not vector genes, could be detected by ddPCR. Single cells are ordered by increasing mean values. (C) Proportion of single cells (%) with predicted vector copy integers determined by Bayesian analysis. The percentage of single cells with five or more vector copies is grouped. Corresponding pVCN from bulk population analysis is indicated at the bottom with the standard deviation, alongside the mean of the single-cell VCN predictions. (D) Comparison between two consecutive microfluidic chips. Two-tail Mann-Whitney test shows a nonsignificant difference between scVCN distributions (p value = 0.9842). (E) Comparison of transduction efficiency values measured with three methods. Flow cytometry indicates the percent of live $ZsGreen^{+}$ cells relative to nontransduced control cells; imaging refers to the number of live green cells on the microfluidic chips relative to the total number of live cells; and PCR-based transduction efficiency derives from the scVCN workflow and is calculated as the number of cells with at least one vector copy relative to the total number of analyzed cells. See also Figure S5.

that offer a more in-depth characterization to support the development of consistently high-quality cell therapies. We have reported here an advanced analytical method for single-cell vector copy distribution and PCR-based transduction efficiency that represents a step change in the ability to evaluate product heterogeneity throughout the drug-development process.

In this study, we have shown that reliable estimation of the integer number of vector copies per single cell is achievable with a rapid and robust ddPCR-based workflow, combined with an in-house Bayesian statistical framework, thus unraveling the underlying heterogeneity during transduction. We have demonstrated that the assay performs consistently across a broad range of VCN values from 1 to 10, which comprises the typical values for clinically relevant lentiviral-based cell therapies. Due to the lack of a suitable reference material, only a proxy for accuracy could be assessed by comparing the mean of the scVCN predictions to an orthogonal measurement of the bulk VCN by ddPCR. By doing so, we have shown that the difference between the two methods is less than or equal to approximately 20%, regardless of the population VCN value. Despite the small number of single cells that can be analyzed by each individual microfluidic chip, the resulting vector copy distributions generated by independent samples from the same population show very similar profiles with no statistical difference, corroborating the reliability of the analysis and the minimal impact of sampling errors. Importantly, this also suggests that the method can be scaled out to increase the throughput of the analysis, which may be relevant if the detection of less frequent events within the population is required. However, whereas increased throughput is often desirable, this must be balanced against the increased workload associated with analysis of multiple microfluidic chips. For instance, analysis of samples with low transduction efficiency (e.g., 20%–30%) could be initially performed using two chips, with the possibility of further scaling out the number of chips if a higher resolution is required.

We have also shown that the scVCN assay is insensitive to ddPCR multiplexing or changes of target primer sequences. This suggests the following: (1) that high-order ddPCR multiplexing strategies may be implemented to reduce the downstream analytical burden, and (2) whereas it was designed to analyze a lentiviral-based product, it can be amenable to other viral and nonviral delivery systems or

transgenes by simply optimizing ddPCR primer sequences complementary to any desired target.

Another parameter required by regulators is transduction efficiency, inferred here as the proportion of cells that have undergone a genetic modification. This is typically measured by flow cytometry, thereby representing the proportion of cells with detectable levels of exogenous protein expression. However, the use of a fluorescent labeling approach is not possible or effective, for instance, when specific antibodies are unavailable, when a protein is ubiquitously expressed, or when mutated proteins are replaced by wild-type ones. Conversely, a PCR-based approach, such as the scVCN assay, overcomes these limitations and offers a rapid and sensitive strategy to distinguish transduced (positive ddPCR signal for both vector and human targets) from nontransduced cells (positive ddPCR signal only for human targets). Notably, other PCR-based approaches are currently employed to measure transduction efficiency, such as a qPCR/ddPCR analysis on clonal populations isolated and expanded from individual cells.[13,18] However, this approach is more variable, nonscalable, and extremely time consuming and is therefore less amenable to supporting rapid developments in the cell therapy field. Another ddPCR-based approach was published that used encapsulation of single cells in oil droplets,[43] yet this method has proved to be difficult to reproduce and extremely variable (unpublished data).

Whereas genotoxicity of lentiviral vectors has improved over the last decades, thanks to the widespread implementation of safer, self-inactivating vectors,[44–48] insertional mutagenesis is nevertheless a risk factor for oncogenesis.[49] Although regulatory agencies acknowledge this risk for gene-modified cell therapies,[9,11,50] they have not yet defined precise rules concerning the acceptable number of vector copies per cell, although the FDA has only provided general guidance that this number should not exceed five copies/cell.[40,51] With the current uncertainty on the long-term biosafety profile for these therapies, the single-cell VCN distribution analysis may provide a more powerful quantification of cell clones with high VCN in the drug products before infusion in patients. In turn, this can increase the predictive power of how vector integrations may influence *in vivo* clonality, persistence, and efficacy of the product, all of which is information that would not be available from conventional population studies. Consequently, we envisage the scVCN assay to enable better process characterization during therapy development by evaluating, for instance, how modulation of critical process parameters may fine tune the resulting scVCN distribution. Similarly, process variability generated, for instance, by a difference in a donor's cells may be investigated in more fine details with this analytical tool, hence providing valuable insights into the transduction process robustness and consistency, which ultimately lead to the generation of high-quality and efficacious products.

In conclusion, with the development of this novel assay, we are pioneering the application of single-cell analysis of gene-modified cell therapies to study product heterogeneity and pinpoint process variability. We envisage the application of this method for the analytical characterization of cell products during process development to support optimized, cost-efficient, and more consistent manufacturing and to monitor the biosafety and critical quality attributes required by regulatory authorities.

MATERIALS AND METHODS

Purification and Cryopreservation of $CD3^+$ Cells

$CD3^+$ cells were purified from healthy donors' Leukopaks received from Hemacare (Northridge, CA, USA). Upon reception, the leukapheresis material was analyzed using the Sysmex pocH-100i Automated Hematology Analyzer (Sysmex, Milton Keynes, UK), diluted with CliniMACS PBS/EDTA buffer (Miltenyi, Bisley, UK), supplemented with 0.5% human AB serum (Seralab, Hayward Heath, UK) and centrifuged at $300 \times g$ for 15 min at room temperature to remove platelets. The $CD3^+$ fraction was purified from the plasma on the CliniMACS Plus instrument (Miltenyi) using the CliniMACS CD4 and CD8 reagents (Miltenyi) anti-CD4 and anti-CD8 monoclonal antibodies conjugated to superparamagnetic iron dextran particles. The viability of the final $CD3^+$ product was assessed using the Vi-CELL XR (Beckman Coulter, High Wycombe, UK) and Nucleocounter NC-200 (ChemoMetec, Allerod, Denmark). The number of $CD45^+$ cells, $CD3^+$ cells, $CD4^+$ cells, and $CD8^+$ cells was evaluated before and after the cell separation on the CliniMACS by flow cytometry on the Fortessa (BD Biosciences, San Jose, CA, USA). The acceptance criteria for the $CD3^+$ cell purification were set as follows: (1) viability over 95% and (2) number of $CD3^+$ cells over 95%. Following purification, a working cell bank of $CD3^+$ cells was cryopreserved in CryoStor CS10 (Sigma, Gillingham, UK) until use.

Transduction and Expansion of $CD3^+$ Cells

Upon thawing, $CD3^+$ T cells were resuspended in X-Vivo 15 (Lonza, Huddersfield, UK), supplemented with 5% human serum (Seralabs, Hayward Heath, UK) and 500 U/mL of Proleukin (Novartis, Camberley, UK) at a cell density of 1×10^6 cells/mL. T cells were activated with Transact (Miltenyi, Bisley, UK) following the manufacturer's recommendations for 6 h before proceeding to viral transduction.

The rLV.EF1.ZsGreen1-9 lentivirus (Clontech, Saint-Germain-en-Laye, France) was thawed on ice and added to the cell suspension. The cell suspension containing the lentiviral vector was seeded into MACS guanosine monophosphate (GMP) Cell Expansion Bags (Miltenyi, Bisley, UK) and incubated for 48 h at 37°C and 5% CO_2. An equal volume of fresh medium containing 1,000 U/mL of Proleukin was added to the bags at days 3 and 5 after transduction. At day 7, the transduction efficiency was evaluated by flow cytometry; thereby, a cell aliquot was labeled with the Live/Dead near-infrared (IR) fixable dye (Life Technologies, Waltham, MA, USA) following the manufacturer's instructions. The remaining culture was resuspended in sterile PBS and processed for FACS or cryopreservation in CryoStor CS10.

Cell Sorting

Transduced T cells were sorted using the FACSAria II (BD Biosciences). Before sorting, cells were labeled with the Live/Dead near-IR fixable dye and resuspended in sterile PBS, supplemented with

2% BSA and 2 mM EDTA (Sigma, Gillingham, UK). The gating strategy was based on forward scatter (FSC) and side scatter (SSC), exclusion of debris and aggregates, and Live/Dead dye exclusion. ZsGreen$^+$ cells were then sorted in sterile PBS, supplemented with 2% BSA and 2 mM EDTA, and cultured in X-Vivo 15 (Lonza), supplemented with 5% human serum (Seralabs) and 500 U/mL of Proleukin (Novartis) at a cell density of 1×10^6 cells/mL for 16–24 h before being cryopreserved in CryoStor CS10 (Sigma) until use. Before processing the cells in the C1 Single-Cell Auto Prep system, the cells were thawed and incubated for 12–16 h at 37°C and 5% CO_2 in complete medium.

Isolation and Processing of Single Cells on the C1 Single-Cell Auto Prep System

The C1 Single-Cell Auto Prep system (Fluidigm, San Francisco, CA, USA) was used to isolate and process transduced T cells on a Fluidigm 10- to 17-μm Single-Cell Open App Integrated Fluidic Circuit (IFC). A custom-made thermal protocol was designed with the C1 Script Builder (Fluidigm) to perform cell capturing, staining, and processing. The complete protocol was divided in three scripts: (1) priming of the IFC; (2) cell loading and staining; and (3) sample prep for lysis and targeted preamplification. The microfluidic chip was loaded using the standard manufacturer's instructions in a segregated pre-PCR room under a dedicated pre-PCR hood and primed on the Fluidigm C1 using the first script. Meanwhile, cells were counted on the Nucleocounter NC-200 and resuspended in PBS at a concentration of 5×10^5 cells/mL. These were then mixed with the C1 Suspension Reagent (Fluidigm) in a 6:4 ratio to create a neutrally buoyant suspension. Once the priming step was completed, the cell suspension was loaded on the microfluidic chip with a Live/Dead staining solution containing calcein blue AM (Thermo Fisher Scientific, Waltham, MA, USA) and ethidium homodimer-1 (Thermo Fisher Scientific) to verify cell viability and number of isolated cells in each capture site, and the second script was run on the Fluidigm C1 system. At the end of this step, the microfluidic chip was imaged on the IN Cell Analyzer 2200 (GE Healthcare Life Sciences, Amersham, UK) using an automated acquisition script that took images in the bright-field, Texas Red excitation channel for ethidium homodimer-1, 4′,6-diamidino-2-phenylindole (DAPI) excitation channel for calcein blue AM, and fluorescein isothiocyanate (FITC) excitation channel for ZsGreen. The capture sites with live single cells were identified for further analysis. For targeted preamplification, custom-made primers were stocked at 20× concentration, corresponding to 6 mM, and the reaction mix was prepared combining 1× PreAmp Master Mix (Fluidigm) with target primers at 0.2× final concentration. All reagents were loaded on the microfluidic chip in a pre-PCR environment, according to the layout generated by the custom script, which was then run on the Fluidigm system on the same day or overnight. Harvesting from the microfluidic chip was performed in a segregated post-PCR room under a dedicated post-PCR hood. The amplified material from each capture site was transferred to a low-bind, 96-well PCR plate, following the layout described by Fluidigm to track the capture-site positions. Before harvesting, the plate was preloaded with 30 μL of Cell DNA Wash Buffer (Fluidigm), and 4 μL of amplified material was added to the plate to obtain 1:8.5 dilution. The diluted material was immediately used in droplet digital PCR.

Extraction of Genomic DNA and Population Analysis

Population VCN was determined using genomic DNA extracted from cell pellets. When this was done in parallel with the single-cell workflow, the remainder of the cell sample not needed for single-cell analysis was pelleted and subjected to gDNA extraction, using the DNeasy Blood and Tissue kit (QIAGEN, Hilden, Germany) and following the manufacturer's instructions. Quantification of the extracted gDNA was determined on the NanoDrop 8000 Spectrophotometer (Thermo Fisher Scientific) to assess stock concentration and quality. 20 ng of gDNA was used in all ddPCR reactions, following the protocol detailed below.

WGA and Targeted Preamplification in Tube

WGA was performed using the commercially available kits: PicoPlex (Rubicon Genomics, Ann Arbor, MI, USA), MALBAC (TATAA, Goteborg, Sweden), and GenomePlex (WGA2; Sigma). All WGA reactions were carried out according to the manufacturer's instructions on gDNA extracted from transduced cells. To minimize the sampling effect, the following amounts of gDNA were used: 30 pg for MALBAC, 10 ng for GenomePlex, and 60 pg for PicoPlex. The resulting amplified DNA was purified using the DNA Clean & Concentrator-5 kit (Zymo Research, Irvine, CA, USA). Cleaned-up products were quantified with the Qubit fluorometer using a double-stranded DNA (dsDNA) BR (broad-range) kit (Invitrogen). Four independent replicates were amplified for each WGA kit. In addition, targeted preamplification was tested using preamp mixes from Fluidigm and Applied Biosystems. Two independent replicates were prepared per mix with 540 pg of the same gDNA used for the WGA kits, according to the manufacturer's instruction. The preamplification program included an initial enzyme activation step, which is 2 min for the Fluidigm preamp and 10 min for the Applied Biosystems preamp, followed by 14 cycles of amplification consisting of 15 s at 95°C and 4 min at 60°C. The products of the WGA and targeted preamplification were assayed in ddPCR using duplex hydrolysis probe-based assays targeting one reference gene (*RPPH1* or *TERT*) and one lentiviral gene (VG1, VG2, or VG3) each. In total, each replicate was assayed six times, with unique ddPCR duplex combinations.

Droplet Digital PCR

Each ddPCR reaction was assembled as a multiplex assay reaction comprising one lentiviral target, labeled with fluorescein amidite (FAM), and either one human reference gene, labeled with ViC/HEX for duplex reactions, or two human reference genes, both labeled with ViC/HEX for triplex reactions. The primers and probes designed in house were purchased from Sigma, whereas the commercial human reference gene assay was purchased from Bio-Rad (in HEX) and Life Technologies (*RPPH1*, *TERT* in ViC). Custom-made primers were stocked at 20× concentration, corresponding to a concentration of 6 μM for the primers and 9 μM for the hydrolysis probe. In duplex reactions, each assay was used at 1× working concentration, whereas

in triplex reactions, one human reference gene was used at 1.2× and the other one at 0.7× working concentration to adjust the fluorescence intensities and obtain optimal cloud separation. The ddPCR reaction mix was prepared containing 1× ddPCR Supermix for Probes without deoxyuridine-triphosphatase (dUTP; Bio-Rad, Watford, UK) and the hydrolysis probe assay in a pre-PCR environment prior to adding 4 μL of the diluted DNA sample in a final reaction volume of 22 μL. The preamplified material from each individual cell was divided appropriately to be used as a template of either six duplex or three triplex ddPCR reactions, allowing multiple measurements from each single cell. All single cells from the same IFC chip were analyzed simultaneously on the same ddPCR plate; therefore, each plate contained only one type of master mix, and multiple plates were generated. Reactions with gDNA from cell pellets and with banks were also prepared and run on the same plate alongside the single-cell material. The assembled ddPCR reactions were pipetted into a semi-skirted and PCR-clean 96-well plate (Bio-Rad) that was then loaded into the Automated Droplet Generator (AutoDG; Bio-Rad) for oil-droplet generation. A Veriti Thermal Cycler (Applied Biosystems) was used for PCR amplification with a ramp rate of 2°C/min (indicated as 60% on Veriti thermocyclers) and the following thermal program: 10 min at 95°C; 30 s at 95°C and 1 min at 62°C for 45 cycles; 10 min at 98°C. Fluorescence amplitude in each droplet was measured on the QX200 Droplet Reader (Bio-Rad) using ddPCR Droplet Reader Oil (Bio-Rad). Data acquisition and analysis were performed with the software package QuantaSoft AP (Bio-Rad). The fluorescence amplitude threshold was manually set closer to the average fluorescence amplitude of the positive droplet cluster. The same threshold was applied to all of the assays of each experiment. Results from single PCR wells were excluded from analysis if one of the following cases was observed: (1) the total number of accepted droplets was <11,000, and (2) the average fluorescence amplitudes of positive or negative droplets were clearly different from those of the other wells on the plate.

Data Analysis

The VCN per diploid genome was calculated as the ratio between the number of vector and reference gene copies, according to the following formula:

$$VCN = 2 \times \frac{vector\ target\ copies}{human\ reference\ target\ copies}.$$

This formula was used for both bulk gDNA and preamplified single material. In both cases, each sample was measured six times with unique combinations generated by six duplex ddPCR reactions or three triplex ddPCR reactions; therefore, six data points per sample were considered for statistical analysis.

Statistical analysis was performed in R (v.3.5.1) or in GraphPad Prism (v.8.3.1). All data points from one experiment were processed together, and quality filtering was applied to remove PCR artifacts. Data points that did not pass the quality filtering were replaced by not applicable (NA), and single cells with more than 3 NAs were removed. Correlation matrices for all measurements were generated using the *cor* function of *stat* package (v.3.5.1), whereas graphs were produced using *corrplot* (v.0.84).

Single-cell VCN predictions were based on Bayesian statistics. In order to estimate the probability of a given copy number, we initially defined a model for the noise in each dataset and assumed for simplicity that the variance was constant regardless of the copy number. To do so, we applied a variance-stabilizing transformation of the data and assumed that the transformed response was an observation of the true copy number under the same transformation plus Gaussian noise with zero mean and constant variance. Prior probabilities were determined by distributing equal probabilities across all possible vector copies in each dataset. The likelihood of each possible copy number was assigned to each single cell by assuming half-normal distribution around each possible copy number with absolute errors (i.e., half-normal distribution for zero copies and normal distribution for copies >0). Normalized posterior probabilities were then inferred by multiplying the obtained likelihood for each copy by the prior probabilities. The maximum likelihood value was then used to identify the best estimate of the vector copy number for each given single cell. Upon averaging the scVCN values, the percent difference (ΔVCN) of the mean of scVCN predictions and the bulk gDNA was calculated with the formula below:

$$\Delta VCN\ (\%) = \frac{\left| pVCN - \overline{scVCN} \right|}{pVCN} \times 100,$$

where $\overline{scVCN}$ is the mean of the single-cell VCN predictions, and pVCN is the bulk gDNA VCN.

SUPPLEMENTAL INFORMATION

Supplemental Information can be found online at https://doi.org/10.1016/j.omtm.2020.04.016.

AUTHOR CONTRIBUTIONS

V.D.C., B.S.-C., and D.M. conceived the work. V.D.C. and I.S. designed the experiments. I.S., V.D.C., M.B., E.H., and E.J.H. performed the experiments. V.D.C. and D.M. wrote the manuscript.

CONFLICTS OF INTEREST

The authors declare no competing interests.

ACKNOWLEDGMENTS

The authors acknowledge Rhys Macown, Nicole Nicholson, and Moira François for experimental support and Heather Turner (Prism Training and Consultancy) for the statistical analysis support, which were paid services by Cell and Gene Therapy Catapult. Gareth A.D. Hardy (Niche Science and Technology) provided writing and editorial support, which were paid services by Cell and Gene Therapy Catapult. The work presented in this study was funded by Innovate UK.

REFERENCES

1. Coppens, D.G.M., de Wilde, S., Guchelaar, H.J., De Bruin, M.L., Leufkens, H.G.M., Meij, P., and Hoekman, J. (2018). A decade of marketing approval of gene and cell-based therapies in the United States, European Union and Japan: An evaluation of regulatory decision-making. Cytotherapy *20*, 769–778.
2. Bravery, C.A., Ball, O., and Robinson, S. (2019). EU market authorisation strategy: lessons from the first 22 ATMP submitted to the EMA. Cell Gene Ther. Insights *5*, 759–791.
3. Yu, T.T.L., Gupta, P., Ronfard, V., Vertès, A.A., and Bayon, Y. (2018). Recent progress in European advanced therapy medicinal products and beyond. Front. Bioeng. Biotechnol. *6*, 130.
4. Alliance for Regenerative Medicine (2019). Q2 2019: Quarterly Global Regenerative Medicine Sector Report, http://alliancerm.org/wp-content/uploads/2019/07/ARM_Q2_2019_Web_FINAL.pdf.
5. Howe, S.J., Mansour, M.R., Schwarzwaelder, K., Bartholomae, C., Hubank, M., Kempski, H., Brugman, M.H., Pike-Overzet, K., Chatters, S.J., de Ridder, D., et al. (2008). Insertional mutagenesis combined with acquired somatic mutations causes leukemogenesis following gene therapy of SCID-X1 patients. J. Clin. Invest. *118*, 3143–3150.
6. Hacein-Bey-Abina, S., Garrigue, A., Wang, G.P., Soulier, J., Lim, A., Morillon, E., Clappier, E., Caccavelli, L., Delabesse, E., Beldjord, K., et al. (2008). Insertional oncogenesis in 4 patients after retrovirus-mediated gene therapy of SCID-X1. J. Clin. Invest. *118*, 3132–3142.
7. Han, H., Braunschweig, U., Gonatopoulos-Pournatzis, T., Weatheritt, R.J., Hirsch, C.L., Ha, K.C.H., Radovani, E., Nabeel-Shah, S., Sterne-Weiler, T., Wang, J., et al. (2017). Multilayered Control of Alternative Splicing Regulatory Networks by Transcription Factors. Mol. Cell *65*, 539–553.e7.
8. Braun, C.J., Boztug, K., Paruzynski, A., Witzel, M., Schwarzer, A., Rothe, M., Modlich, U., Beier, R., Göhring, G., Steinemann, D., et al. (2014). Gene therapy for Wiskott-Aldrich syndrome—long-term efficacy and genotoxicity. Sci. Transl. Med. *6*, 227ra33.
9. Aiuti, A., Cossu, G., de Felipe, P., Galli, M.C., Narayanan, G., Renner, M., Stahlbom, A., Schneider, C.K., and Voltz-Girolt, C. (2013). The committee for advanced therapies' of the European Medicines Agency reflection paper on management of clinical risks deriving from insertional mutagenesis. Hum. Gene Ther. Clin. Dev. *24*, 47–54.
10. European Medicines Agency, Science Medicines Health (2018). Guideline on quality, non-clinical and clinical aspects of medicinal products containing genetically modified cells. EMA/CAT/GTWP/671639/2008 Rev. 1, https://www.ema.europa.eu/en/documents/scientific-guideline/draft-guideline-quality-non-clinical-clinical-aspects-medicinal-products-containing-genetically_en.pdf.
11. National Archives and Records Administration (2006). Guidance for Industry Gene Therapy Clinical Trials—Observing Subjects for Delayed Adverse Events; Availability. Federal Register. A notice from the Food and Drug Administration, November 28, 2006, https://www.federalregister.gov/documents/2006/11/28/E6-20129/guidance-for-industry-gene-therapy-clinical-trials-observing-subjects-for-delayed-adverse-events.
12. Baum, C., Düllmann, J., Li, Z., Fehse, B., Meyer, J., Williams, D.A., and von Kalle, C. (2003). Side effects of retroviral gene transfer into hematopoietic stem cells. Blood *101*, 2099–2114.
13. Kustikova, O.S., Wahlers, A., Kühlcke, K., Stähle, B., Zander, A.R., Baum, C., and Fehse, B. (2003). Dose finding with retroviral vectors: correlation of retroviral vector copy numbers in single cells with gene transfer efficiency in a cell population. Blood *102*, 3934–3937.
14. Ritchie, D.S., Neeson, P.J., Khot, A., Peinert, S., Tai, T., Tainton, K., Chen, K., Shin, M., Wall, D.M., Hönemann, D., et al. (2013). Persistence and efficacy of second generation CAR T cell against the LeY antigen in acute myeloid leukemia. Mol. Ther. *21*, 2122–2129.
15. Zhang, B., Metharom, P., Jullie, H., Ellem, K.A., Cleghorn, G., West, M.J., and Wei, M.Q. (2004). The significance of controlled conditions in lentiviral vector titration and in the use of multiplicity of infection (MOI) for predicting gene transfer events. Genet. Vaccines Ther. *2*, 6.
16. Burke, C.J., and Zylberberg, C. (2019). Sources of Variability in Manufacturing of Cell Therapeutics. Regen. Eng. Transl. Med. *5*, 332–340.
17. Charrier, S., Ferrand, M., Zerbato, M., Précigout, G., Viornery, A., Bucher-Laurent, S., Benkhelifa-Ziyyat, S., Merten, O.W., Perea, J., and Galy, A. (2011). Quantification of lentiviral vector copy numbers in individual hematopoietic colony-forming cells shows vector dose-dependent effects on the frequency and level of transduction. Gene Ther. *18*, 479–487.
18. Urbinati, F., Campo Fernandez, B., Masiuk, K.E., Poletti, V., Hollis, R.P., Koziol, C., Kaufman, M.L., Brown, D., Mavilio, F., and Kohn, D.B. (2018). Gene Therapy for Sickle Cell Disease : A Lentiviral Vector Comparison Study. Hum. Gene Ther. *29*, 1153–1166.
19. Fehse, B., Kustikova, O.S., Bubenheim, M., and Baum, C. (2004). Pois(s)on–it's a question of dose. Gene Ther. *11*, 879–881.
20. Saliba, A.E., Westermann, A.J., Gorski, S.A., and Vogel, J. (2014). Single-cell RNA-seq: advances and future challenges. Nucleic Acids Res. *42*, 8845–8860.
21. Hwang, B., Lee, J.H., and Bang, D. (2018). Single-cell RNA sequencing technologies and bioinformatics pipelines. Exp. Mol. Med. *50*, 96.
22. Peterson, V.M., Zhang, K.X., Kumar, N., Wong, J., Li, L., Wilson, D.C., Moore, R., McClanahan, T.K., Sadekova, S., and Klappenbach, J.A. (2017). Multiplexed quantification of proteins and transcripts in single cells. Nat. Biotechnol. *35*, 936–939.
23. Shahi, P., Kim, S.C., Haliburton, J.R., Gartner, Z.J., and Abate, A.R. (2017). Abseq: Ultrahigh-throughput single cell protein profiling with droplet microfluidic barcoding. Sci. Rep. *7*, 44447.
24. Stoeckius, M., Hafemeister, C., Stephenson, W., Houck-Loomis, B., Chattopadhyay, P.K., Swerdlow, H., Satija, R., and Smibert, P. (2017). Simultaneous epitope and transcriptome measurement in single cells. Nat. Methods *14*, 865–868.
25. Spitzer, M.H., and Nolan, G.P. (2016). Mass Cytometry: Single Cells, Many Features. Cell *165*, 780–791.
26. Ramani, V., Deng, X., Qiu, R., Gunderson, K.L., Steemers, F.J., Disteche, C.M., Noble, W.S., Duan, Z., and Shendure, J. (2017). Massively multiplex single-cell Hi-C. Nat. Methods *14*, 263–266.
27. Cusanovich, D.A., Daza, R., Adey, A., Pliner, H.A., Christiansen, L., Gunderson, K.L., Steemers, F.J., Trapnell, C., and Shendure, J. (2015). Multiplex single cell profiling of chromatin accessibility by combinatorial cellular indexing. Science *348*, 910–914.
28. Argelaguet, R., Mohammed, H., Clark, S.J., Stapel, L.C., Krueger, C., Kapourani, C.-A., Xiang, Y., Hanna, C., Smallwood, S., Ibarra-Soria, X., et al. (2019). Single cell multi-omics profiling reveals a hierarchical epigenetic landscape during mammalian germ layer specification. bioRxiv. https://doi.org/10.1101/519207.
29. Yin, Y., Jiang, Y., Lam, K.G., Berletch, J.B., Disteche, C.M., Noble, W.S., Steemers, F.J., Camerini-Otero, R.D., Adey, A.C., and Shendure, J. (2019). High-Throughput Single-Cell Sequencing with Linear Amplification. Mol. Cell *76*, 676–690.e10.
30. McConnell, M.J., Lindberg, M.R., Brennand, K.J., Piper, J.C., Voet, T., Cowing-Zitron, C., Shumilina, S., Lasken, R.S., Vermeesch, J.R., Hall, I.M., and Gage, F.H. (2013). Mosaic copy number variation in human neurons. Science *342*, 632–637.
31. Hindson, B.J., Ness, K.D., Masquelier, D.A., Belgrader, P., Heredia, N.J., Makarewicz, A.J., Bright, I.J., Lucero, M.Y., Hiddessen, A.L., Legler, T.C., et al. (2011). High-throughput droplet digital PCR system for absolute quantitation of DNA copy number. Anal. Chem. *83*, 8604–8610.
32. Pinheiro, L.B., Coleman, V.A., Hindson, C.M., Herrmann, J., Hindson, B.J., Bhat, S., and Emslie, K.R. (2012). Evaluation of a droplet digital polymerase chain reaction format for DNA copy number quantification. Anal. Chem. *84*, 1003–1011.
33. Wang, Y., Bergelson, S., and Feschenko, M. (2018). Determination of lentiviral infectious titer by a novel droplet digital PCR method. Hum. Gene Ther. Methods *29*, 96–103.
34. Lin, H.-T., Okumura, T., Yatsuda, Y., Ito, S., Nakauchi, H., and Otsu, M. (2016). Application of Droplet Digital PCR for Estimating Vector Copy Number States in Stem Cell Gene Therapy. Hum. Gene Ther. Methods *27*, 197–208.
35. Huang, J.T., Liu, Y.J., Wang, J., Xu, Z.G., Yang, Y., Shen, F., Liu, X.H., Zhou, X., and Liu, S.M. (2015). Next generation digital PCR measurement of hepatitis B virus copy number in formalin-fixed paraffin-embedded hepatocellular carcinoma tissue. Clin. Chem. *61*, 290–296.
36. Strain, M.C., Lada, S.M., Luong, T., Rought, S.E., Gianella, S., Terry, V.H., Spina, C.A., Woelk, C.H., and Richman, D.D. (2013). Highly precise measurement of HIV DNA by droplet digital PCR. PLoS ONE *8*, e55943.

37. Lillsunde Larsson, G., and Helenius, G. (2017). Digital droplet PCR (ddPCR) for the detection and quantification of HPV 16, 18, 33 and 45 - a short report. Cell Oncol. (Dordr.) *40*, 521–527.

38. Deprez, L., Corbisier, P., Kortekaas, A.M., Mazoua, S., Beaz Hidalgo, R., Trapmann, S., and Emons, H. (2016). Validation of a digital PCR method for quantification of DNA copy number concentrations by using a certified reference material. Biomol Detect. Quantif. *9*, 29–39.

39. Brunetto, G.S., Massoud, R., Leibovitch, E.C., Caruso, B., Johnson, K., Ohayon, J., Fenton, K., Cortese, I., and Jacobson, S. (2014). Digital droplet PCR (ddPCR) for the precise quantification of human T-lymphotropic virus 1 proviral loads in peripheral blood and cerebrospinal fluid of HAM/TSP patients and identification of viral mutations. J. Neurovirol. *20*, 341–351.

40. Zhao, Y., Stepto, H., and Schneider, C.K. (2017). Development of the First World Health Organization Lentiviral Vector Standard: Toward the Production Control and Standardization of Lentivirus-Based Gene Therapy Products. Hum. Gene Ther. Methods *28*, 205–214.

41. Bersenev, A., and Kili, S. (2018). Management of 'out of specification' commercial autologous CAR-T cell products. Cell Gene Ther. Insights *4*, 1051–1058.

42. Better, M., Chiruvolu, V., and Sabatino, M. (2018). Overcoming Challenges for Engineered Autologous T Cell Therapies. Cell Gene Ther. Insights *4*, 173–186.

43. Igarashi, Y., Uchiyama, T., Minegishi, T., Takahashi, S., Watanabe, N., Kawai, T., Yamada, M., Ariga, T., and Onodera, M. (2017). Single Cell-Based Vector Tracing in Patients with ADA-SCID Treated with Stem Cell Gene Therapy. Mol. Ther. Methods Clin. Dev. *6*, 8–16.

44. Dull, T., Zufferey, R., Kelly, M., Mandel, R.J., Nguyen, M., Trono, D., and Naldini, L. (1998). A third-generation lentivirus vector with a conditional packaging system. J. Virol. *72*, 8463–8471.

45. Yu, S.F., von Rüden, T., Kantoff, P.W., Garber, C., Seiberg, M., Rüther, U., Anderson, W.F., Wagner, E.F., and Gilboa, E. (1986). Self-inactivating retroviral vectors designed for transfer of whole genes into mammalian cells. Proc. Natl. Acad. Sci. USA *83*, 3194–3198.

46. Naldini, L., Trono, D., and Verma, I.M. (2016). Lentiviral vectors, two decades later. Science *353*, 1101–1102.

47. Kotterman, M.A., Chalberg, T.W., and Schaffer, D.V. (2015). Viral Vectors for Gene Therapy: Translational and Clinical Outlook. Annu. Rev. Biomed. Eng. *17*, 63–89.

48. Booth, C., Gaspar, H.B., and Thrasher, A.J. (2016). Treating Immunodeficiency through HSC Gene Therapy. Trends Mol. Med. *22*, 317–327.

49. Sadelain, M. (2004). Insertional oncogenesis in gene therapy: how much of a risk? Gene Ther. *11*, 569–573.

50. European Medicines Agency, Science Medicines Health (2013). Reflection paper on management of clinical risks deriving from insertional mutagenesis. EMA/CAT/190186/2012, https://www.ema.europa.eu/en/documents/scientific-guideline/reflection-paper-management-clinical-risks-deriving-insertional-mutagenesis_en.pdf.

51. Hollyman, D., Stefanski, J., Przybylowski, M., Bartido, S., Borquez-Ojeda, O., Taylor, C., Yeh, R., Capacio, V., Olszewska, M., Hosey, J., et al. (2009). Manufacturing validation of biologically functional T cells targeted to CD19 antigen for autologous adoptive cell therapy. J. Immunother. *32*, 169–180.

OMTM, Volume *17*

Supplemental Information

Vector Copy Distribution at a Single-Cell Level Enhances Analytical Characterization of Gene-Modified Cell Therapies

Ilaria Santeramo, Marta Bagnati, Emily Jane Harvey, Enas Hassan, Beata Surmacz-Cordle, Damian Marshall, and Vincenzo Di Cerbo

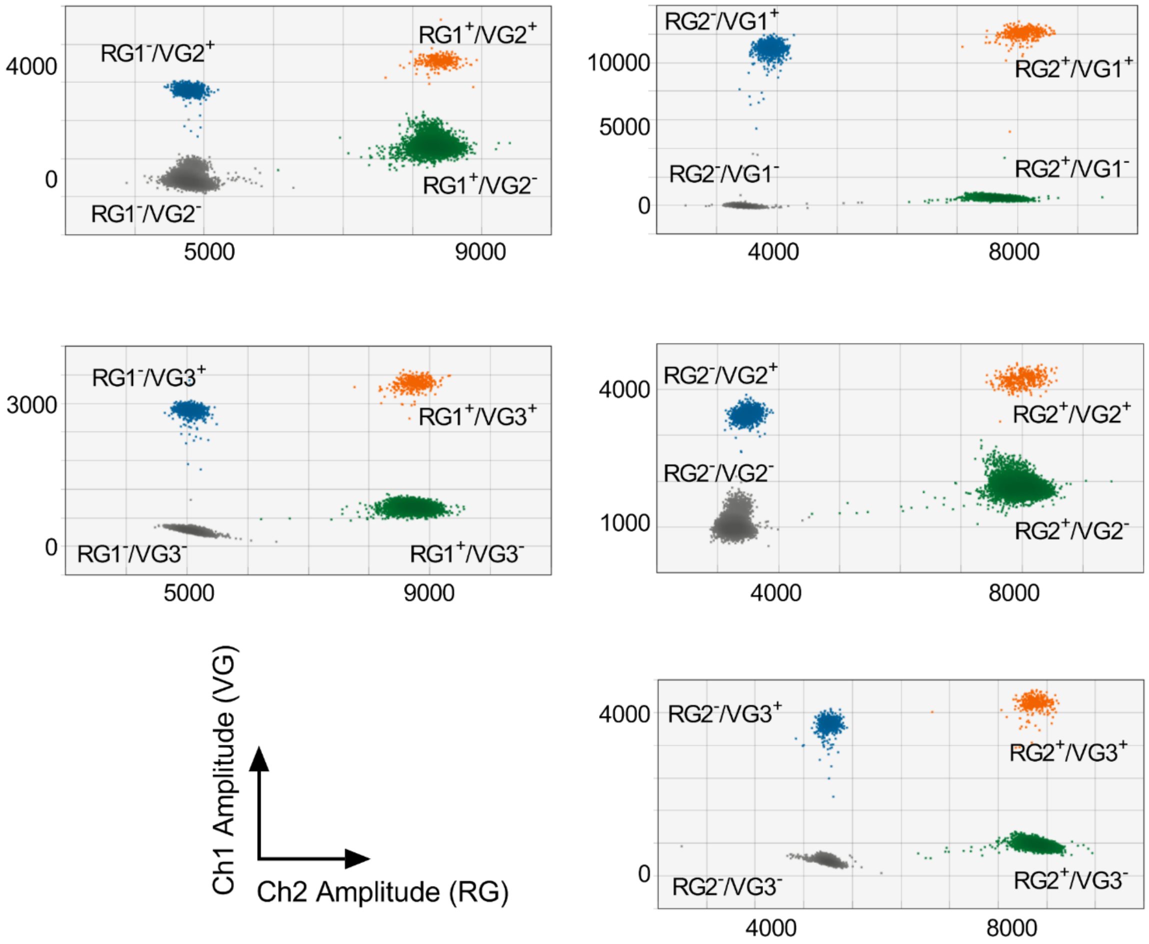

Figure S1 Representative 2D plot of duplex ddPCR reactions using different combinations of vector or human reference genes (see also Figure 1). Single positive droplets are shown in green for human targets (RG) and in blue for viral targets (VG), whereas double positive droplets are in orange and double negatives are in grey.

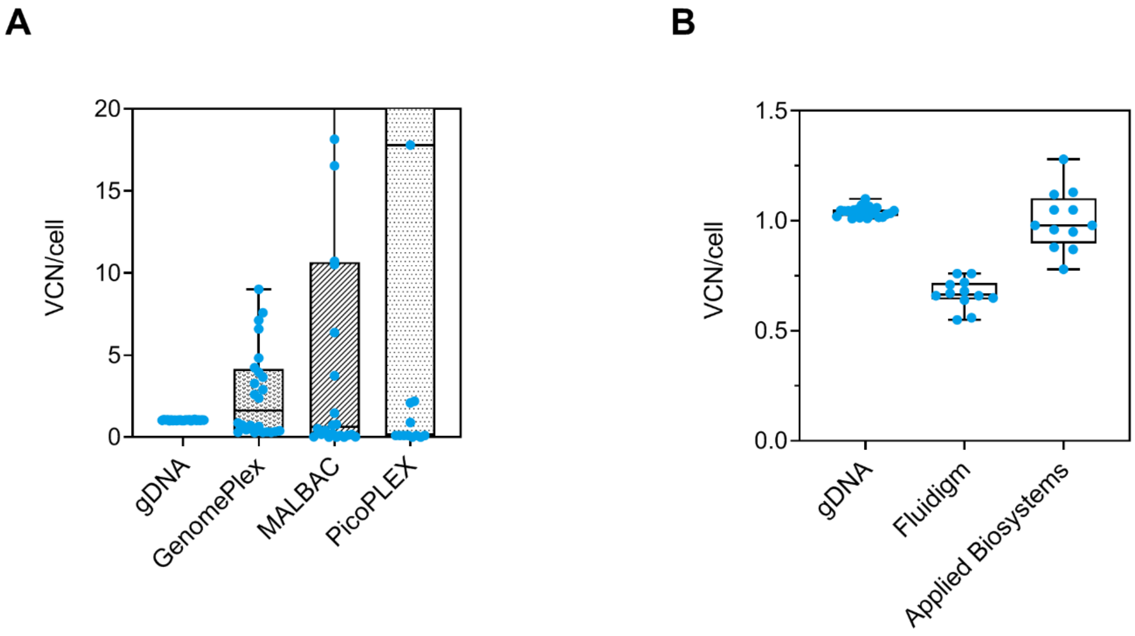

Figure S2 VCN analysis comparing WGA and targeted pre-amplification (see also Figure 2). (A) Summary of the vector copy number measured from each WGA reaction with the indicated commercial kits. Non-amplified genomic DNA is shown as control. Each boxplot (n=4, 24 data points) is represented with median and whiskers spanning from min to max value. For clarity, y-axis has been limited to 20 VCN/cell for an optimal visualisation of the data points closer to the gDNA positive control. (B) Summary of the vector copy number measured from each targeted pre-amplification reaction with the indicated commercial master mix. Non-amplified genomic DNA is shown as a control. Each boxplot (n=2, 12 data points) is represented with median and whiskers spanning from min to max value.

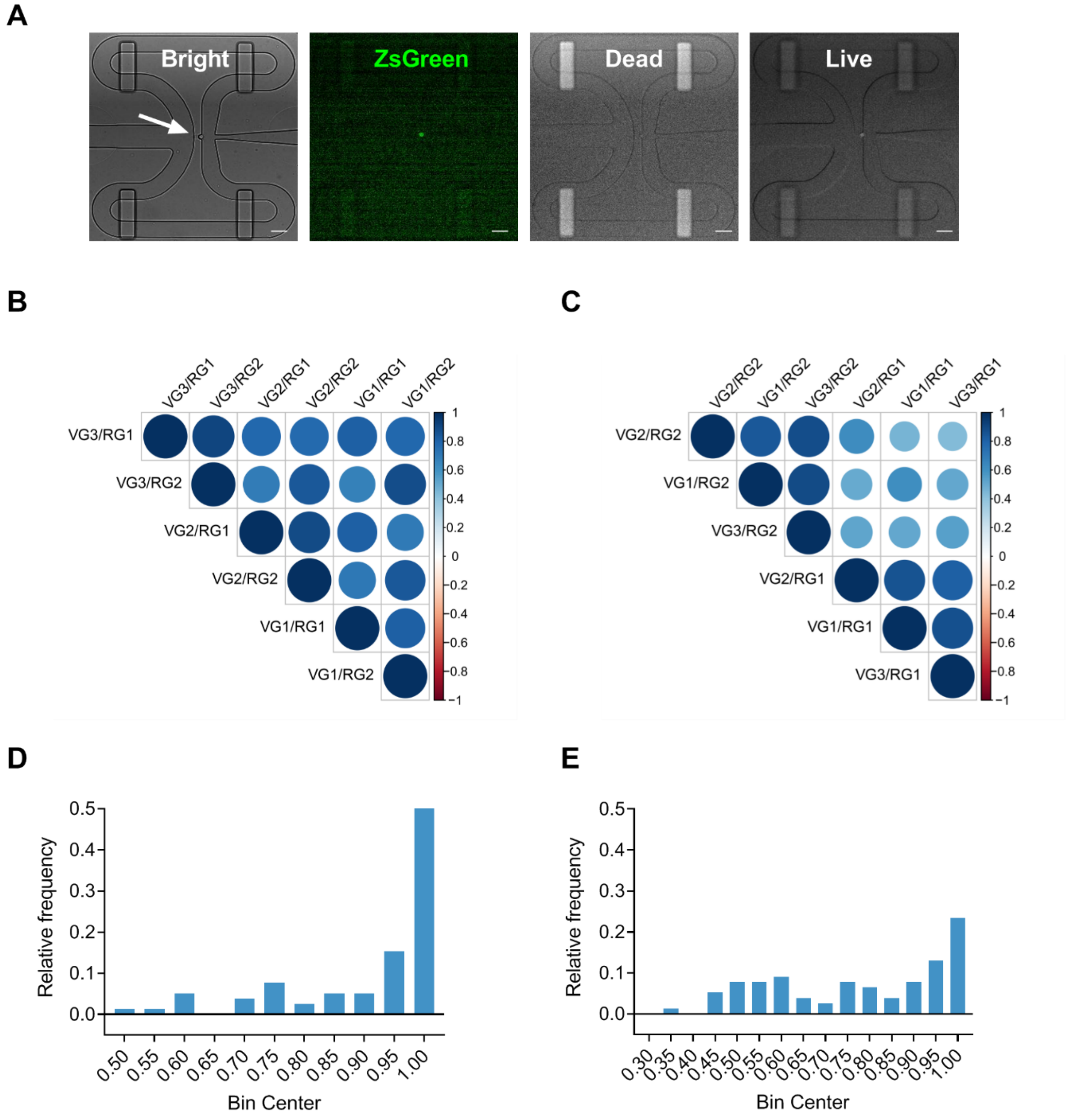

Figure S3 scVCN assay controls (see also Figure S3). (A) Representative images of one capture site in the Fluidigm OpenApp microfluidic chip. The arrow in the brightfield image indicates the capture site. Green signal derives from fluorescent ZsGreen protein. Live/dead detect the presence of calcein blue AM for live cells or ethidium homodimer-1 for dead cells (negative in this representative single cell). Scale bar = 60 µm. (B-C) Pairwise Spearman correlation between all duplex combinations on the "low VCN" (B) or the "high VCN" samples (C). Dark blue indicates positive correlation, whereas red indicates anti-correlation and the legend on the right shows the corresponding correlation coefficients. (D-E) Frequency distribution of highest posterior probabilities from the Bayesian analysis corresponding to the scVCN predictions with the highest likelihood. The probabilities for the low or high VCN samples are shown in (D) or (E), respectively. Bin width is 0.05.

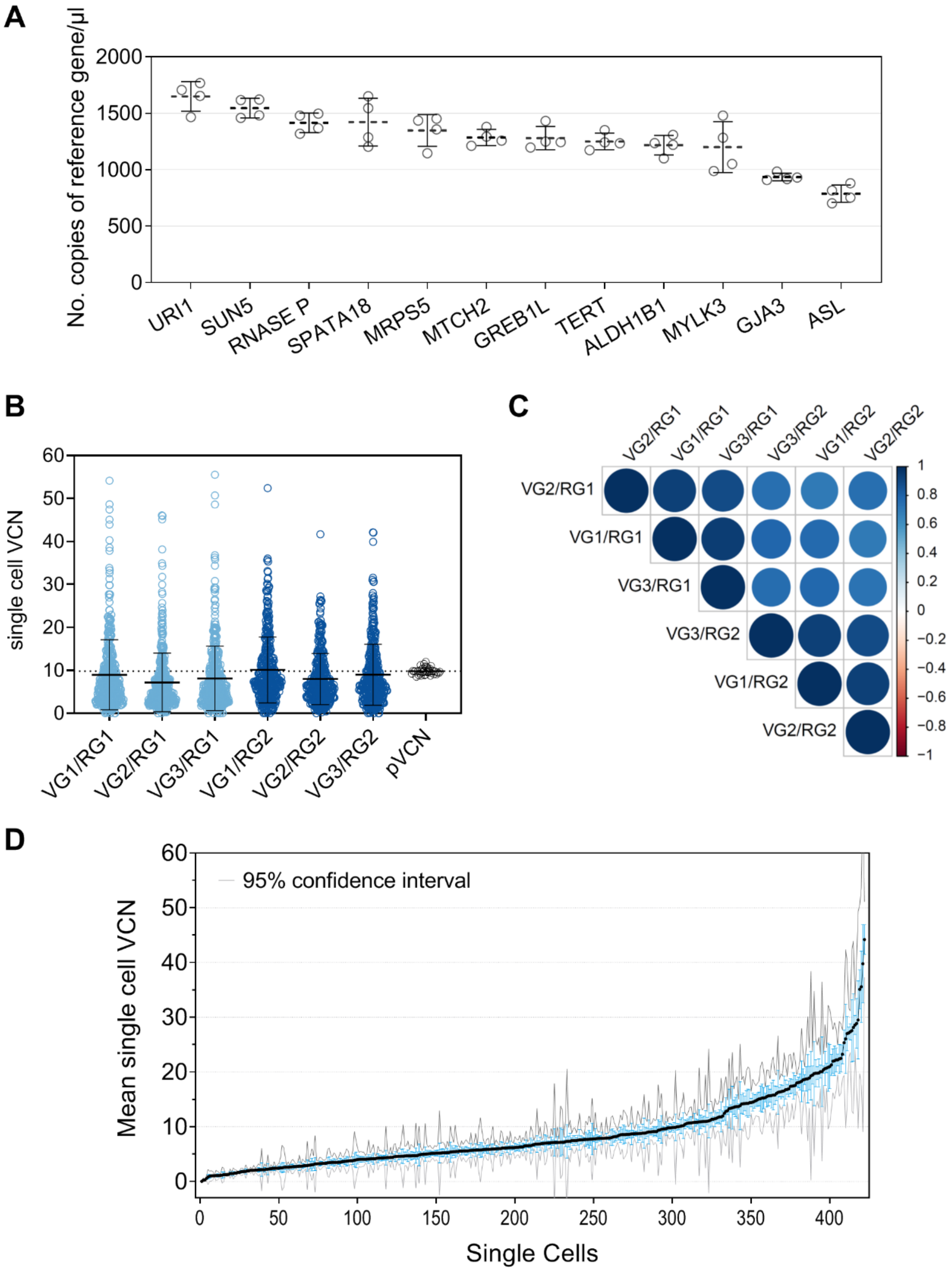

Figure S4 scVCN assay optimisation and scale out (see also Figure 4). (A) Absolute number of copies per µL of reaction of twelve reference genes tested on the genomic DNA equivalents of 80 cells pre-amplified with Fluidigm pre-amp mix (n=4). Each boxplot is represented with mean and standard deviation. (B) Single-cell VCN values from three triplex ddPCR assays (one VG and two RG in each reaction) generate six unique VG/RG measurements. scVCN values are in light blue for RG1 and in dark blue for RG2. pVCN from identical triplex ddPCR on bulk gDNA is shown in grey. Each boxplot is represented with mean and standard deviation. (C) Pairwise Spearman correlation between the six scVCN combinations across all six microfluidics chips. Dark blue indicates positive correlation, whereas red indicates anti-correlation and the legend on the right shows the corresponding correlation coefficients. (D) The mean of the six scVCN combinations is shown for each single cell and represented with standard error and 95% confidence interval. Single cells are ordered by increasing mean values.

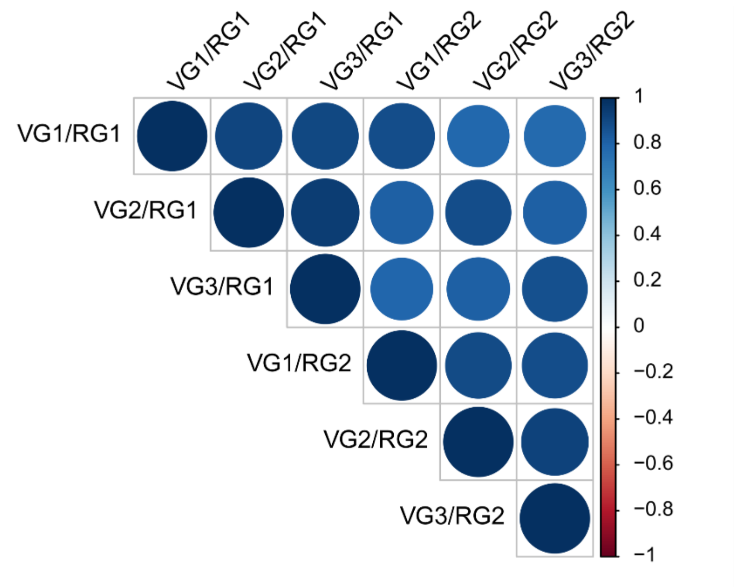

Figure S5. scVCN assay control for a non-sorted sample (see also Figure 5). Pairwise Spearman correlation between the six scVCN combinations. Dark blue indicates positive correlation, whereas red indicates anti-correlation and the legend on the right shows the corresponding correlation coefficients.

Table S1 VCN result summary of pre-amplification methods on gDNA (see also Figure 2).

	**Mean**	**SD**	**CV**
**GenomePlex**	2.66	2.71	102%
**MALBAC**	15.20	41.45	273%
**PicoPLEX**	918.70	2440.00	266%
**Fluidigm pre-amp mix**	0.67	0.07	10%
**Applied Biosystems pre-amp mix**	1.00	0.13	13%
**gDNA (non-amplified)**	1.04	0.02	2%
